# Allelic Imbalance in Regulation of *ANRIL* through Chromatin Interaction at 9p21 Endometriosis Risk Locus

**DOI:** 10.1371/journal.pgen.1005893

**Published:** 2016-04-07

**Authors:** Hirofumi Nakaoka, Aishwarya Gurumurthy, Takahide Hayano, Somayeh Ahmadloo, Waleed H Omer, Kosuke Yoshihara, Akihito Yamamoto, Keisuke Kurose, Takayuki Enomoto, Shigeo Akira, Kazuyoshi Hosomichi, Ituro Inoue

**Affiliations:** 1 Division of Human Genetics, Department of Integrated Genetics, National Institute of Genetics, Mishima, Shizuoka, Japan; 2 Department of Obstetrics and Gynecology, Niigata University Graduate School of Medical and Dental Sciences, Chuo-ku, Niigata, Japan; 3 Department of Obstetrics and Gynecology, Nippon Medical School, Bunkyo-ku, Tokyo, Japan; 4 Department of Bioinformatics and Genomics, Graduate School of Medical Sciences, Kanazawa University, Kanazawa, Ishikawa, Japan; Yale School of Medicine, UNITED STATES

## Abstract

Genome-wide association studies (GWASs) have discovered numerous single nucleotide polymorphisms (SNPs) associated with human complex disorders. However, functional characterization of the disease-associated SNPs remains a formidable challenge. Here we explored regulatory mechanism of a SNP on chromosome 9p21 associated with endometriosis by leveraging “allele-specific” functional genomic approaches. By re-sequencing 1.29 Mb of 9p21 region and scrutinizing DNase-seq data from the ENCODE project, we prioritized rs17761446 as a candidate functional variant that was in perfect linkage disequilibrium with the original GWAS SNP (rs10965235) and located on DNase I hypersensitive site. Chromosome conformation capture followed by high-throughput sequencing revealed that the protective G allele of rs17761446 exerted stronger chromatin interaction with *ANRIL* promoter. We demonstrated that the protective allele exhibited preferential binding affinities to TCF7L2 and EP300 by bioinformatics and chromatin immunoprecipitation (ChIP) analyses. ChIP assays for histone H3 lysine 27 acetylation and RNA polymerase II reinforced the enhancer activity of the SNP site. The allele specific expression analysis for eutopic endometrial tissues and endometrial carcinoma cell lines showed that rs17761446 was a *cis*-regulatory variant where G allele was associated with increased *ANRIL* expression. Our work illuminates the allelic imbalances in a series of transcriptional regulation from factor binding to gene expression mediated by chromatin interaction underlie the molecular mechanism of 9p21 endometriosis risk locus. Functional genomics on common disease will unlock functional aspect of genotype-phenotype correlations in the post-GWAS stage.

## Author Summary

A large number of variants associated with human complex diseases have been discovered by genome-wide association studies (GWASs). These discoveries have been anticipated to be translated into the definitive understanding of disease pathogeneses; however, functional characterization of the disease-associated SNPs remains a formidable challenge. Here we explored regulatory mechanism of a variant on chromosome 9p21 associated with endometriosis, a common gynecological disorder. By scrutinizing linkage disequilibrium structure and DNase I hypersensitive sites across the risk locus, we prioritized rs17761446 as a candidate causal variant. The results of our “allele-specific” functional genomic approaches sheds light on regulatory mechanisms underlying 9p21 endometriosis risk locus, in which preferential bindings of TCF7L2 and its coactivator EP300 to the protective G allele of rs17761446 lead to stronger chromatin interaction with the promoter of *ANRIL*, which in turn activate transcription of the non-coding RNA. Motivated by the fact that TCF7L2 was a key transcription factor of Wnt signaling pathway, we postulated that the induction of Wnt signaling activated expression levels of *ANRIL* and cell cycle inhibitors, *CDKN2A*/*2B*. Functional genomics on common disease will unlock functional aspect of genotype-phenotype correlations in the post-GWAS stage.

## Introduction

With the advent of genome-wide association studies (GWASs), a large number of single nucleotide polymorphisms (SNPs) associated with human complex diseases have been discovered [1]. The findings from GWASs have provided previously unsuspected biological pathways relevant to the diseases [2] such as complement system in age-related macular degeneration [3] and autophagy in Crohn’s disease [4]. Furthermore, the annotation of the disease-associated SNPs to plausible susceptibility genes and pathways can be translated into drug discovery and repositioning [5]. However, functional characterization of the identified SNPs remains a formidable challenge [6].

Most of the identified SNPs are located on intergenic regions and introns rather than coding regions [7], suggesting that these non-coding SNPs are associated with the disease risk through regulation of expression levels of nearby genes. This argument has been supported by the fact that the disease-associated SNPs were overrepresented in expression quantitative trait loci (eQTL) of genes [8–10]. The ENCODE and Roadmap Epigenomics projects have explored functional elements across the human genome in a wide variety of cell types [11, 12]. The integrative approaches for linking the regulatory elements with the SNPs identified by GWASs highlighted the importance of DNase I hypersensitive sites (DHSs) in the genotype-phenotype correlations [11, 13], which signify accessible open chromatin regions. A large proportion of the SNPs identified by GWASs or tagged by them were located on DHSs in cell types relevant to the corresponding diseases and overlapped transcription factor (TF) recognition sequences [14]. These systematic analyses present a plausible model in which the disease-associated SNPs alter activities of *cis*-regulatory elements including promoters, enhancers, insulators, and silencers. There is a growing body of evidence supporting this view from functional genomic studies in coronary artery disease (CAD) [15], cholesterol levels [16], obesity [17], type 2 diabetes [18], cancers [19–21], erythroid traits [22], and pigmentation traits [23–25]. It has been also reported that mutations on *cis*-regulatory elements cause severe developmental disorders showing a Mendelian pattern of inheritance [26].

Current GWAS platforms contain now up to 1M SNPs, which are designed to efficiently surrogate known common variants covering the human genome. Therefore, there is a possibility that the SNPs identified by GWASs are merely surrogate markers for causal variants. Although a comprehensive list of SNPs in the associated loci has been genotyped, the association signals for many correlated SNPs within a linkage disequilibrium (LD) block are statistically indistinguishable. Under these circumstances, it can be a straightforward way to prioritize candidate causal SNPs by searching for SNPs that are in strong LD with the SNPs identified by the GWASs and located on DHSs in cell types relevant to the disease for subsequent functional genomic approaches.

Endometriosis is a common gynecological disorder that is characterized by the presence of uterine endometrial tissue outside the normal location. The prevalence in women of reproductive age is estimated to be 6–10% [27]. Endometriosis is a risk factor for several subtypes of ovarian cancer [28]. GWASs of endometriosis in Japanese and European populations have identified several susceptibility loci [29–33]. Uno and colleagues reported that rs10965235, locating in an intron of *ANRIL* (antisense non-coding RNA in the INK4 locus or *CDKN2B-AS1*) on chromosome 9p21, was significantly associated with endometriosis in Japanese population [29]. rs10965235 has been reported to be most strongly associated with the risk of endometriosis in Japanese population (per allele odds ratio of 1.44) but rare or absent in European descent populations [29]. This association was replicated in an independent GWAS in Japanese [30]. Meta-analysis of European and Japanese GWAS data sets identified rs1537377 at 49kb downstream of *ANRIL* [32]. rs1537377 is common both in European descent and Japanese populations and associated with modest increase of the risk for endometriosis (per allele odds ratio of 1.15) [32]. These two SNPs on 9p21 were shown to be independent association signals [32], but their functional roles have not been characterized.

Here we investigated regulatory mechanism of the endometriosis risk locus on 9p21. Coupled with target re-sequencing of 9p21 region and DNase-seq data from the ENCODE project, we prioritized candidate causal variants that were in perfect LD with the SNP identified by the original GWAS and located on DHSs. Subsequent functional genomic approaches revealed that the SNP site functioned as a *cis*-regulatory element of *ANRIL* through allele-specific long-range chromatin interaction driven by preferential bindings of TCF7L2 and EP300. Furthermore, we demonstrated that expressions of *ANRIL* and *CDKN2A/2B* were closely associated via Wnt signaling pathway. These results suggest that the 9p21 risk locus is involved in the development of endometriosis by modulating the expression level of *ANRIL* and *CDKN2A/2B*.

## Results

### Prioritizing candidate causal variants of the 9p21 locus via target re-sequencing and DNase-seq data

**Fig 1:**
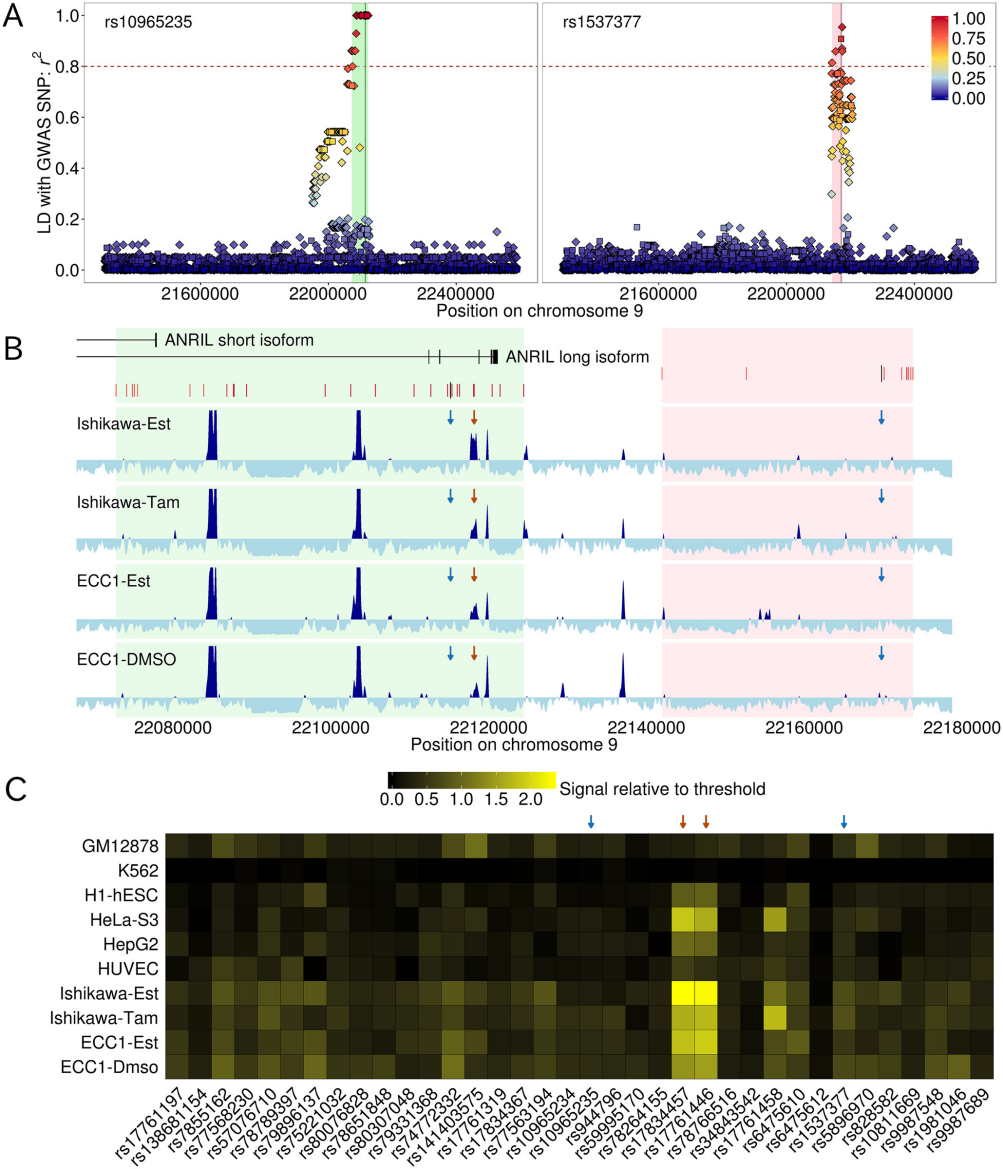
Prioritization of candidate causal variants via target re-sequencing and DNase-seq data. A) Distribution of variants exhibiting strong LD (*r*^2^ > 0.8) with two endometriosis-associated SNPs (rs10965235 and rs1537377). SNPs and indels are coded by diamonds and squares, respectively. The positions of rs10965235 and rs1537377 are shown as vertical lines. The intervals encompassing all the variants showing strong LD with rs10965235 and rs1537377 are highlighted by light green and pink shades, respectively. B) DHSs in endometrial carcinoma cell lines across the two intervals. The transcript structures of *ANRIL* and the sites of the variants that are in strong LD are depicted. The densities of aligned reads from DNase-seq estimated by F-Seq are plotted. DHSs in which the densities of aligned reads significantly surpassing the threshold are represented by dark blue. Locations in which aligned reads are depleted are depicted by light blue. The positions of the SNPs identified by GWASs (rs10965235 and rs1537377) and candidate causal SNPs (rs17834457 and rs17761446) are highlighted by blue and red arrows, respectively. C) DNase-seq signals at the variant sites showing strong LD. Signals are represented as relative values to the threshold determined by F-Seq. If the relative signal surpasses 1.0, the corresponding variant site coincides with significant DHS.

In order to assemble a comprehensive set of genetic variants on 9p21 region, we re-sequenced 1.29 Mb interval encompassing two endometriosis-associated SNPs (rs10965235 and rs1537377) in 48 Japanese individuals with the average depth of 186.6 (S1–S3 Figs). We detected 4,215 single nucleotide variants (SNVs) and 664 insertions and deletions (indels) with a high degree of confidence (S4–S6 Figs). We compiled a list of variants that were in strong LD with rs10965235 or rs1537377 (Fig 1A and S1 Table). There were 24 SNPs and two indels exhibiting strong LD with rs10965235 (*r*^2^ > 0.8). Among them, 16 SNPs and two indels were in perfect LD (*r*^2^ = 1.0), and therefore the association cannot be distinguished. For rs1537377, seven SNPs and one indel were detected. The intervals containing the variants exhibiting strong LD with rs10965235 and rs1537377 are located in the 3’ region of *ANRIL* (Fig 1B). We confirmed that rs10965235 and rs1537377 were in weak LD each other (*r*^2^ = 0.02), and in weak and moderate LD with the 9p21 SNPs associated with other diseases (S7 Fig), indicating that these two associations were independent endometriosis-specific signals.

We explored DHSs to prioritize candidate causal variants. Among the ENCODE cell lines with DNase-seq data, we focused on endometrial carcinoma cell lines (Ishikawa and ECC1) as a cell type potentially relevant to endometriosis because endometrial carcinoma arose from the endometrium. Additionally, we examined six cell lines, consisting of lymphoblastoid (GM12878), chronic myeloid leukemia (K562), H1 embryonic stem cell (H1-hESC), hepatoblastoma (HepG2), cervical cancer (HeLa-S3), and umbilical vein epithelial cell (HUVEC) with high priority in the ENCODE project (Tiers 1 and 2). We detected a distinct set of DHSs across 101 kb interval containing all the variants showing strong LD with rs10965235 and rs1537377 (chr9: 22072730–22173676) in these cell lines (Figs 1B and S8). The two SNPs identified by the original GWAS did not lie on DHSs in the endometrial carcinoma cell lines and other cell lines analyzed (Fig 1B and 1C); therefore, we excluded these two variants from candidate. We identified a site harboring two SNPs (rs17761446 and rs17834457) where significant DNase-seq signals were consistently detected in the endometrial carcinoma cell lines (Fig 1B and 1C). rs17761446 and rs17834457 are closely located (76 bp apart) and in perfect LD with rs10965235 (*r*^2^ = 1.0). The DHS harboring rs17761446 and rs17834457 seems to be cell-type specific open chromatin site (Ishikawa, ECC1, and HeLa-S3) rather than ubiquitous one (Fig 1C). We could not identify any SNPs that were in strong LD with rs1537377 and coincided on DHSs. Therefore, we focused on the DHS containing rs17761446 and rs17834457 for subsequent functional genomic studies.

### Allele-specific chromatin interaction between DHS containing candidate SNPs and *ANRIL* promoter

**Fig 2:**
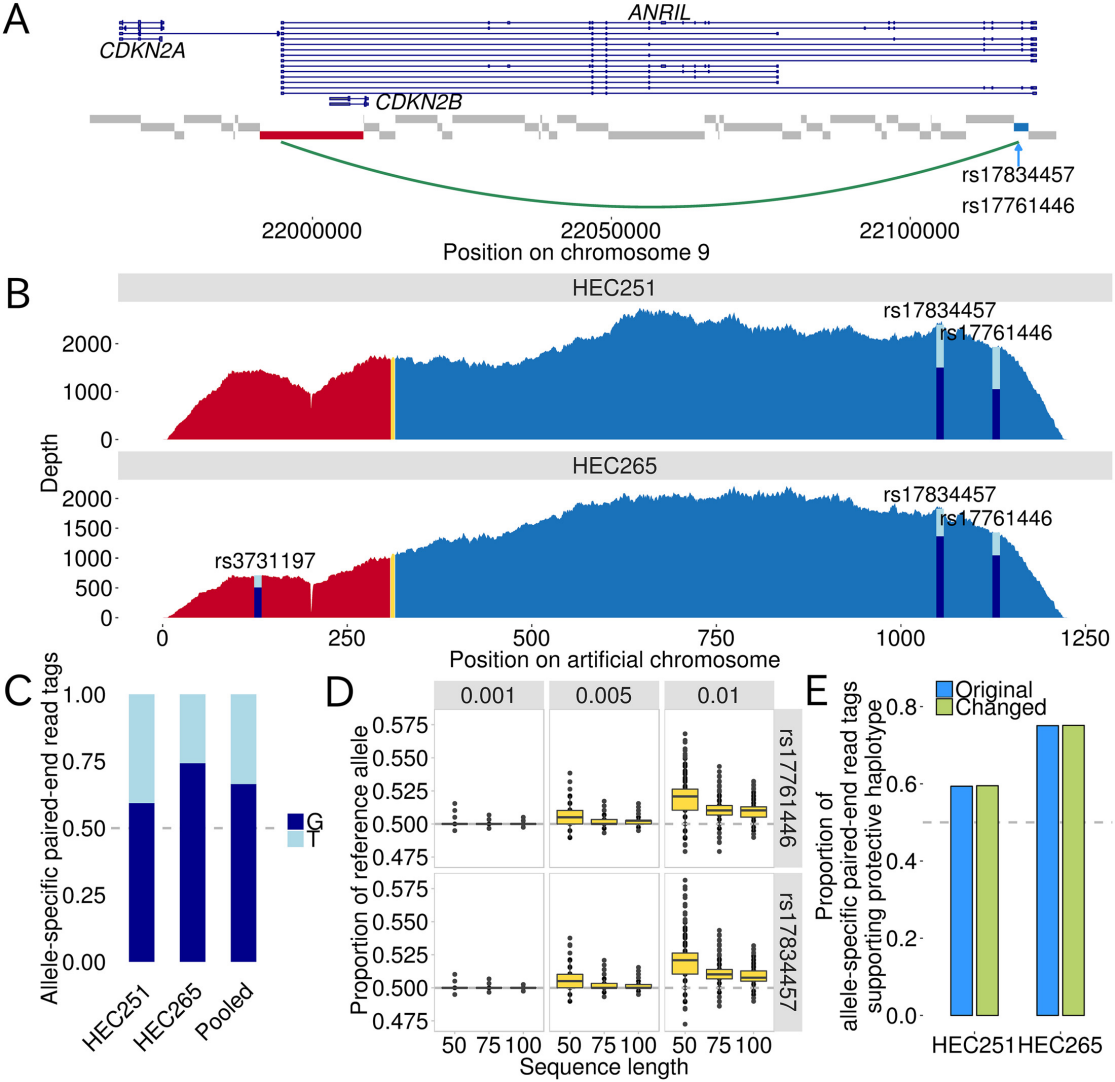
AS3C-seq for the detection of allele-specific chromatin interaction. A) The interacting fragments between the candidate causal SNPs (rs17834457 and rs17761446) and the promoter of *ANRIL* are color-coded by blue and red, respectively. *Hin*dIII fragments are depicted by gray rectangles. The alternative transcript structures *ANRIL*, *CDKN2A*, and *CDKN2B* are represented. B) Plots of coverage depth for AS3C-seq libraries from two endometrial carcinoma cell lines (HEC251 and HEC265). The fragments between the candidate causal SNPs (rs17834457 and rs17761446) and the promoter of *ANRIL* are color-coded by blue and red, respectively. The ligation junction (i.e., restriction enzyme recognition sequence of *Hin*dIII, AAGCTT) is shown by yellow. For heterozygous SNP sites, the proportions of allele-specific paired-end read tag counts supporting the alleles on the risk and protective haplotypes are shown by light blue and dark blue, respectively. It can be seen that a region within the fragment containing *ANRIL* promoter shows reduced depth of coverage. This seems to be caused by sequence or alignment errors because the region corresponds to a homopolymer stretch with 15 consecutive adenines (see also S12 Fig), indicating that this does not largely affect our results. C) Proportions of allele-specific paired-end read tag counts supporting the risk and protective haplotypes tagged by T and G alleles of rs17761446, respectively. Results for the two cell lines and for pooled data are shown. D) Simulation study for evaluating mapping bias. Simulation scenarios considering different sequence lengths (50bp, 75bp, and 100bp) and different error rates for each sequenced base (0.01, 0.005, and 0.001). Box plots are based on 10,000 replicates. rs17761446 and rs17834457 are apart from 76bp and in perfect linkage disequilibrium (*r*^2^ = 1); therefore, we consider the haplotype structure of these two variants in the simulation scenario in which sequence length is 100bp. E) Sensitivity analysis for evaluating mapping bias. In sensitivity analyses, we aligned reads from AS3C-seq to two types of sequences that were different in the base at the SNP site: the “original” and “changed” sequences have “reference” and “alternative” alleles, respectively. Note that the significant result from AS3C-seq exhibited increased proportion of alternative allele.

The DHS harboring rs17761446 and rs17834457 located on an intron of long isoforms of *ANRIL* (Fig 1B), approximately 123, 143, and 109 kb apart from the transcription start site of *ANRIL*, *CDKN2A*, and *CDKN2B*, respectively. Therefore, we hypothesized that the DHS was a distal regulatory element contacting with the promoter of *ANRIL*, or *CDKN2A/2B* through chromatin looping interaction. We investigated long-range interactions using chromosome conformation capture (3C) assay [34]. As a first step, we examined chromatin loops formed between the restriction fragment containing the candidate causal SNPs (rs17761446 and rs17834457) and the consecutive restriction fragments around *ANRIL*, *CDKN2A*, and *CDKN2B* (S2 Table). The consistent PCR amplifications of the interacting fragments between the candidate causal SNPs and the promoter of *ANRIL* were observed in replicated experiments from HEC251 and HEC265 (S9A Fig). Additionally, Sanger sequencing for the PCR product confirmed that the sequence for the ligation junction between fragments between the candidate causal SNPs and the promoter of *ANRIL* was successfully identified, supporting the presence of the chromatin interaction (S9B Fig). Therefore, we focused attention on the chromatin interaction between the SNP site and the promoter of *ANRIL* (Fig 2A).

We developed a novel 3C-based approach to detect allele-specific chromatin interactions (AS3C-seq) (Methods and S10 and S11 Figs). Briefly, the AS3C-seq quantifies a difference in chromatin interaction frequencies between two alleles of a SNP in the sequence reads of the fragmented 3C PCR product by using cell lines that are heterozygous for the SNP. In this study, we used HEC251 and HEC265 that were heterozygous for the candidate SNPs (rs17761446 and rs17834457). The substantial numbers of reads (>1,000) were aligned to the ligation junction, supporting the presence of the chromatin interaction between *ANRIL* promoter and the candidate causal SNPs (Figs 2B and S11 and S12).

rs17834457 and rs17761446 are in perfect LD and therefore constitute two haplotypes; risk and protective haplotypes. The proportions of the read tags supporting the protective haplotype over the total mapped tag counts were 59.4% and 75.1% in HEC251 and HEC265, respectively (Fig 2B). These were significantly deviated from the expected proportion of 50% (*P* < 10^−16^; binomial test). Additionally, HEC265 was heterozygote for a SNP (rs3731197) within the fragment containing the promoter of *ANRIL*. The re-sequencing of the 1.29 Mb interval and haplotype phasing showed that the C allele of rs3731197 resided on the protective haplotype in HEC265. The AS3C-seq result showed the proportion of the C allele of rs3731197 was similar to those at the two candidate causal SNPs (71.7%; *P* < 10^−16^) (Fig 2B). The consistent results in both of the interacting fragments suggest the quantification of allele-specific read counts in the AS3C-seq is reliable. In the pooled data set from HEC251 and HEC265, the proportion of allele-specific read counts supporting the protective haplotype was 66.5% (*P* < 10^−16^; likelihood ratio test), indicating that the protective haplotype tagged by the G allele of rs17761446 forms two times greater chromatin interactions with the promoter of *ANRIL* compared to the risk haplotype (Fig 2C).

One reservation on allele specific functional genomic studies based on next generation sequencing technologies is mapping bias [35]. In order to evaluate mapping bias on our AS3C-seq analysis, we performed simulation and sensitivity analyses (Methods and S10C Fig). According to the results of the simulation and sensitivity analyses, we confirmed that mapping bias does not affect our result (Fig 2D and 2E; Methods).

### rs17761446 alters the binding affinities of TCF7L2 and its coactivator EP300

**Fig 3:**
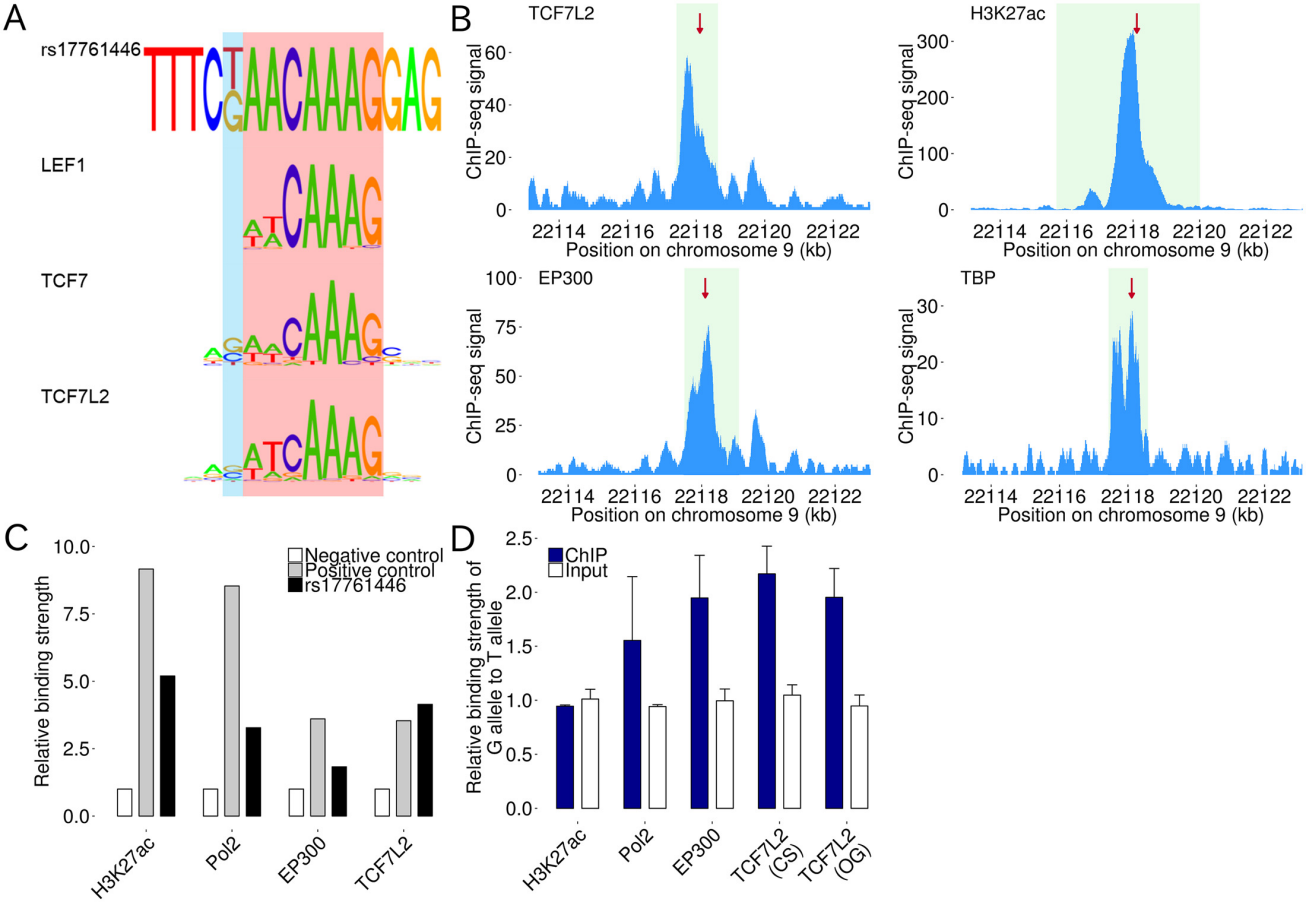
rs17761446 alters binding affinities of TCF7L2 and EP300. A) Alignments of consensus motifs of TCF/LEF family to sequence surrounding rs17761446. Core motif of HMG class of TFs are highlighted by red. The position of rs17761446 is highlighted by blue. B) ChIP-seq signals for TCF7L2, EP300, H3K27ac, and TBP in HeLa-S3 cells from ENCODE project. Plots are created for 10 kb around rs17761446. Significant peak regions are highlighted by light green. C) ChIP assays for TCF7L2, EP300, H3K27ac, and RNA polymerase II at rs17761446 site in HEC251 cells. *MYC* promoter and α satellite repeat element were used as positive and negative control regions, respectively. The enrichment of the normalized binding affinity for the candidate SNP site or positive control region was normalized by dividing by that for negative control region. D) Binding strength of the G allele relative to the T allele of rs17761446 in HEC251 cells. Data represents the mean and standard deviation of triplicated ChIP assays. Pol2, RNA polymerase II; TCF7L2 (CS) and TCF7L2 (OG), TCF7L2 antibodies by Cell Signaling Technology and OriGene Technologies, respectively.

We examined whether rs17761446 and rs17834457 overlapped with consensus TF binding site within the DHS by bioinformatics analysis (S13 and S14 Figs and Methods). As a result, we found that rs17761446 lay adjacent to the core motif of HMG (high mobility group) class of TFs including TCF/LEF and SOX families (Figs 3A and S15). According to the position weight matrices (PWMs) in the HOCOMOCO database [36], rs17761446 lies within the binding motifs of TCF7 and TCF7L2, which favors the G allele over the T allele (Fig 3A). Additionally, the G allele matches with the consensus binding motif of drosophila TCF family member (AAGATCAAAGG) [37].

Chromatin immunoprecipitation (ChIP)-seq data from the ENCODE project has demonstrated the binding of TCF7L2 to rs17761446 site in HeLa-S3, which harbors a DHS in this SNP site (Figs 1C and 3B). Additionally, the bindings of EP300 (a coactivator for LEF/TCF family of transcription factors) and H3K27ac (a histone modification characteristic of active enhancer) in HeLa-S3 cells indicate that the SNP site facilitates a distal enhancer activity (Fig 3B). Additionally, colocalization of TATA-binding protein (TBP) with the enhancer marks at rs17761446 seems to reinforce the presence of the enhancer-promoter interaction (Fig 3B). On the other hand, we could not find consistent results between predicted binding motifs overlapping with rs17834457 and evidence of bindings from the ENCODE ChIP-seq analyses. Accordingly, we focused attention on rs17761446, and then demonstrated the bindings of TCF7L2, EP300, H3K27ac, and RNA polymerase II to the SNP site in HEC251 cells by ChIP assays (Fig 3C).

We quantified allele-specific factor bindings at rs17761446 using TaqMan-based allelic discrimination assay. The allelic ratios in immunoprecipitated and input chromatins were determined by fitting their log2 transformed VIC/FAM ratios to the standard curve constructed from DNA mixtures with known allelic ratios (S16 and S17 Figs). As a result, we detected significant allelic imbalances in the occupancies of TCF7L2 in two distinct antibodies (Cell Signaling, *P* = 0.019; OriGene, *P* = 0.012; combined *P* = 5.7×10^−5^) and EP300 (*P* = 7.0×10^−3^) (S17 Fig). These factors selectively bound to the G allele over the T allele of rs17761446, in which the occupancies of the G allele reached ~70% (TCF7L2 Cell Signaling, 68.3%; OriGene, 66.0%; and EP300, 65.7%) (S18 Fig). This indicates that the G allele exerts 2.06- and 1.95-fold greater binding affinities to TCF7L2 and EP300, respectively (Fig 3D). RNA polymerase II strongly bound to the G allele by a factor of 1.55 (Fig 3D), although the allelic imbalance did not reach to significance level (*P* = 0.14). On the other hand, H3K27ac did not exhibit allele-specific bindings (*P* = 0.50).

### Allele specific expression of *ANRIL* in the haplotype tagged by rs17761446

**Fig 4:**
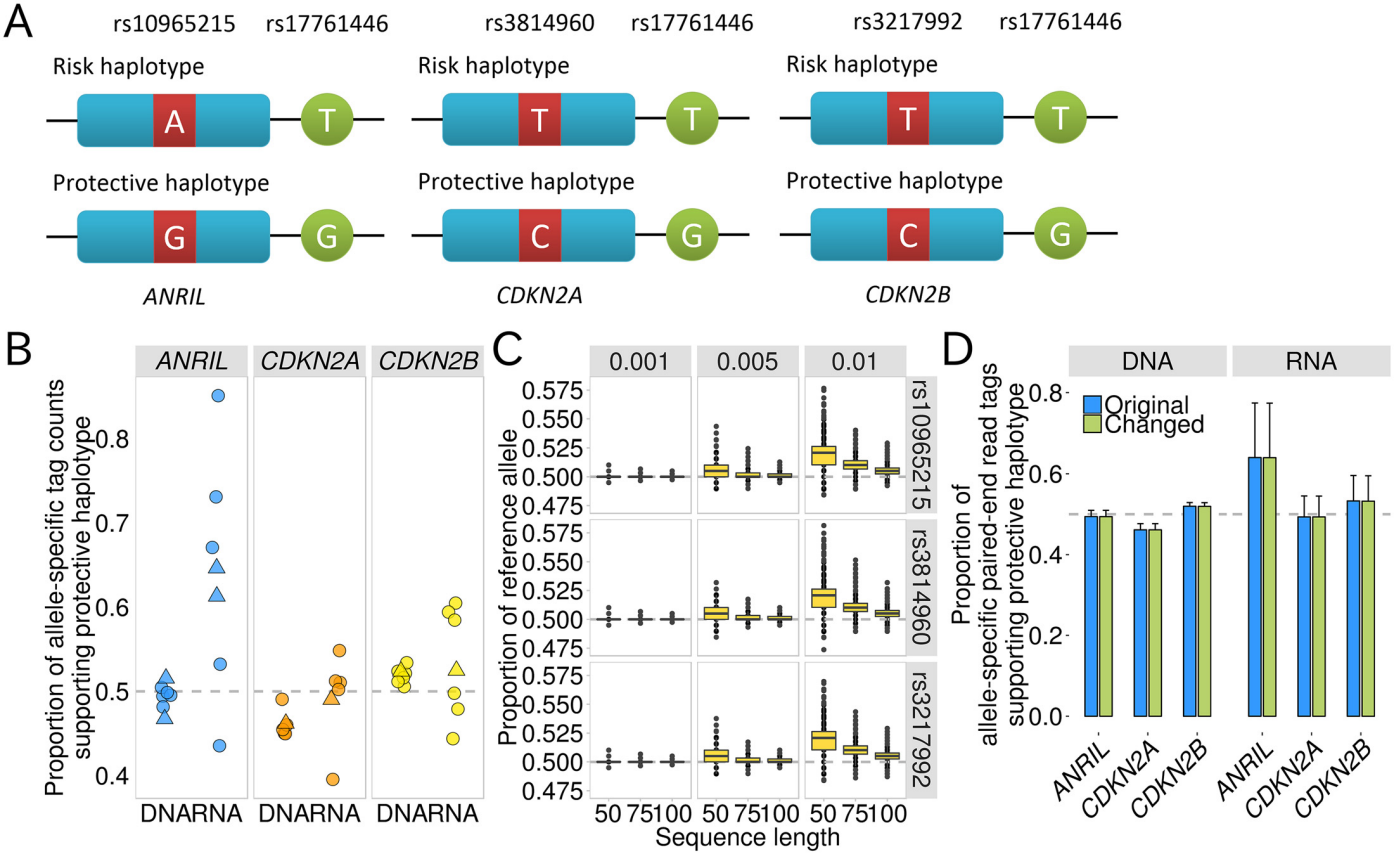
ASE analyses of rs17761446 for 9p21 genes. A) Haplotypes constructed with candidate causal SNP rs17761446 and SNPs within transcribed region of 9p21 genes (*ANRIL*, rs10965215; *CDKN2A*, rs3814960; and *CDKN2B*, rs3217992). B) Proportions of allele-specific paired-end tag counts supporting the protective haplotype in genome DNA and RNA samples from eutopic endometrial tissues (circles) and endometrial carcinoma cell lines (triangles). C) Simulation study for evaluating mapping bias. Simulation scenarios considering different sequence lengths (50bp, 75bp, and 100bp) and different error rates for each sequenced base (0.01, 0.005, and 0.001). Box plots are based on 10,000 replicates. D) Sensitivity analysis for evaluating mapping bias. In sensitivity analyses, we aligned reads from ASE to two types of sequences that were different in the base at the SNP site: the “original” and “changed” sequences have “reference” and “alternative” alleles, respectively. For each bar plot, mean and standard deviation are shown. Note that the significant result from *ANRIL* exhibited increased proportion of alternative allele.

We evaluated whether rs17761446 worked as a *cis*-regulatory element of *ANRIL*, *CDKN2A*, and *CDKN2B* with the allele specific expression (ASE) analysis using eutopic endometrial tissues and endometrial carcinoma cell lines. We determined the haplotype phases with re-sequencing data for 9p21 region, and then screened the samples that were heterozygous both for rs17761446 and SNPs within transcribed regions of 9p21 genes with the same haplotypes (Fig 4A and Methods). The allele-specific paired-end read tag counts were measured by the deep sequencing of the reverse transcription PCR products. Genomic DNAs from the corresponding samples were also deeply sequenced as control. The average of the paired-end read tag counts was greater than 20,000. While the average proportion of the G allele of rs10965215, which was located on an exon of *ANRIL* and resided on the protective haplotype tagged by the G allele of rs17761446, was 49.4% over the genomic DNA samples, the paired-end read tags supporting the protective haplotype were over-represented in the RNA samples (on average 64.0%). The difference between the genomic DNA and RNA samples was statistically significant (*P* = 0.026) (Fig 4B). This result indicates that rs17761446 was a *cis*-regulatory element of *ANRIL* and the protective allele was associated with 1.78-fold higher expression of *ANRIL* than the risk allele. On the other hand, the two alleles were not differentially transcribed in *CDKN2A* (*P* = 0.20) and *CDKN2B* (*P* = 0.61) (Fig 4B). These results seem to be consistent with the result that the G allele of rs17761446 showed stronger chromatin interaction with the promoter of *ANRIL* rather than *CDKN2A/2B*. We confirmed that the result of our ASE analysis was robust against mapping bias by using simulation and sensitivity analyses (Fig 4C and 4D; Methods).

### Induction of Wnt signaling activates expression levels of 9p21 genes

According to the results showing that the *cis*-regulatory element at rs17761446 regulated *ANRIL* expression and a key TF for canonical Wnt signaling, TCF7L2 [38], differentially bound to the SNP alleles, we hypothesized that the induction of Wnt signaling changed expression level of *ANRIL*. Wnt signaling was induced by CHIR99021 (CHIR) in HEC251 cell (S19 Fig). CHIR is a small molecule inhibitor of GSK3, and therefore can activate Wnt pathway through decreased phosphorylation and subsequent stabilization of β-catenin [38, 39]. We examined the effects of CHIR treatment in HEC251 cells on the expression of long and short isoforms of *ANRIL* (S20A Fig). Compared to cells treated with vehicle only (DMSO), the CHIR treatment significantly increased the expression levels of long and short isoforms of *ANRIL* by 33.2% (*P* = 5.7×10^−3^) and 53.9% (*P* = 1.6×10^−3^), respectively (S20 Fig). The effect was greater on the short isoform of *ANRIL*. We confirmed the increase in overall *ANRIL* transcripts using the primer pair amplifying both long and short isoforms (29.4%, *P* = 9.4×10^−4^) (S20B Fig). *ANRIL* is thought to regulate the INK4/ARF tumor suppressor locus [40, 41]; therefore, we examined expressions of two *CDKN2A* transcripts (*p16*^*INK4A*^ and *p14*^*ARF*^) and one *CDKN2B* transcript (*p15*^*INK4B*^). The expression levels of *p16*^*INK4A*^ and *p15*^*INK4B*^ significantly increased by 38.7% (*P* = 5.5×10^−6^) and 35.7% (*P* = 5.0×10^−4^), respectively, with the CHIR treatment (S20B Fig). On the other hand, *p14*^*ARF*^ did not change (*P* = 0.24). This result implies that the induction of Wnt signaling activates *ANRIL* expression, which in turn leads to increased expressions of G1 cell-cycle inhibitors, *p16*^*INK4A*^ and *p15*^*INK4B*^. In order to present a more direct evidence linking the candidate SNP and the change in *ANRIL* expression by inducing Wnt signaling via CHIR treatment, we evaluated whether degree of allelic imbalance of *ANRIL* expression increased after inducing Wnt signaling via CHIR treatment. For this purpose, we performed ASE analysis of *ANRIL* by using CHIR- and DMSO-treated endometrial carcinoma cell line (HEC251). Although we replicated ASE of *ANRIL* where protective haplotype was associated with increased expression (CHIR, *P* = 2.8×10^−4^; DMSO, *P* = 7.8×10^−4^; one sample *t*-test), there was no difference in ASE between two treatments (*P* = 0.83; Welch’s *t*-test) (S21 Fig).

## Discussion

The major challenge in the post-GWAS stage is to elucidate the functional aspect of genotype-phenotype correlations, i.e., molecular mechanisms and biological pathways, affected by the identified genetic variants. The post-GWAS analysis is essential for the translation of genetic knowledge to clinical application. Functional studies focusing only on the SNPs identified by GWASs may not be promising because the identified SNPs are most likely surrogate markers for causal variants. The information about regulatory elements distributed across the risk locus provided by the ENCODE project can be a powerful tool to prioritize putative causal variants. Additionally, the regulatory mechanism modulated by candidate causal variant must be experimentally unraveled. In this study, we addressed these two steps to elucidate transcriptional regulation of the endometriosis-associated variants on chromosome 9p21.

We demonstrated that integrative analysis of the LD profile created by re-sequencing of the risk locus with information about regulatory elements from DNase-seq data provided by the ENCODE consortium was effective to prioritize putative causal variants. As a consequence, we found that the SNP identified by the original GWAS did not coincide with DHSs in the endometrial carcinoma cell lines and other representative cell types. On the other hand, we identified two SNPs (rs17761446 and rs17834457) showing strong DNase-seq signals in the endometrial carcinoma and HeLa cells (Fig 1).

We leveraged “allele-specific” functional genomic approaches to full advantage for the identification of allelic imbalances in distinct stages of transcriptional regulation including binding affinities of TF and its coactivator, chromatin interactions, and gene expressions. The advantage of allele-specific functional genomic approaches is that the use of tissues or cell lines that were heterozygote for a SNP of interest allows to directly compare functional difference between the two alleles of the SNP under the same cellular environment [42]. To the best of our knowledge, only a few studies addressed allelic differences in the frequencies of chromatin interactions between two alleles of causal SNPs [21–23]. In this study, we developed a method to detect allele-specific chromatin interactions (AS3C-seq) (S10 Fig). The AS3C-seq utilizes the next-generation sequencing technology to quantify the difference in chromatin interaction frequencies between the two alleles of a SNP of interest. By using AS3C-seq, we detected an allelic imbalance in the frequencies of chromatin interactions between candidate causal SNP and the promoter of *ANRIL*. The AS3C-seq showed that a DHS in an intron of *ANRIL* containing two candidate causal SNPs formed chromatin looping with the promoter of *ANRIL* and the interaction frequencies differed according to the SNP alleles (Fig 2). One of these two SNPs (rs17761446) overlapping with binding motifs of HMG class of TFs including TCF/LEF and SOX families was shown to alter the binding affinities to TCF7L2 and EP300 (Fig 3). The differential bindings of TCF7L2 and EP300 between the two alleles of rs17761446 seem to result in the observed allelic imbalance in chromatin interactions between the SNP site and the promoter of *ANRIL*, because the DNA bending properties of TCF/LEF lead to the formation of DNA loops favoring the concentration of distant transcriptional complexes [43] and their coactivator EP300 facilitates as a bridge between DNA-binding TFs and basal transcription machinery such as TFIIB and TBP [44]. The ASE analysis showed the allelic imbalance in the transcription of *ANRIL* between the SNP alleles by using eutopic endometrial tissues and endometrial carcinoma cell lines, suggesting that rs17761446 work as a *cis*-acting eQTL of *ANRIL* (Fig 4). The results of this study sheds light on regulatory mechanisms underlying 9p21 endometriosis risk locus, in which preferential bindings of TCF7L2 and its coactivator EP300 to the protective G allele of rs17761446 lead to stronger chromatin interaction with the promoter of *ANRIL*, which in turn activate transcription of the non-coding RNA.

Although we initially focused on binding of TCF7L2 according to consistent results between the motif search and the ENCODE ChIP-seq analyses, SOX family members can be a good candidate for a regulator of the enhancer ability at the 9p21 endometriosis risk locus. Indeed, we found that SOX4 strongly bound to the candidate SNP site (S22 Fig). Co-localization of SOX4 with TCF7L2 at the SNP site seems to be intriguing, because SOX4 has been reported to regulate Wnt signaling by direct binding to TCF family members and β-catenin [45]. According to the result of motif search, other SOX family members (SOX5, SOX9, SOX13, SOX15, and SRY) were also predicted to bind to the SNP site (S13 and S15 Figs). It has been reported that some SOX factors work as agonist and promote Wnt signaling, others act as antagonist [46]. Therefore, further analyses for evaluating bindings of a set of SOX family members at the SNP site together with allele-specific binding analyses may be useful to uncover regulatory mechanism of the 9p21 endometriosis risk locus.

Motivated by the fact that TCF7L2 is a key TF in canonical Wnt pathway [46], we demonstrated that expression level of *ANRIL* was increased by inducing Wnt signal by CHIR treatment. At the same time, we found increased expressions of *CDKN2A* (*p16*^*INK4A*^) and *CDKN2B* (*p15*^*INK4B*^) (S20B Fig). As shown in Fig 4, rs17761446 did not exert *cis*-regulatory effects on *CDKN2A* and *CDKN2B*. Therefore, it is speculated that *ANRIL* modulates expressions of *CDKN2A* and *CDKN2B* through *trans*-regulation, though we could not rule out the possibility that other regulatory mechanisms could account for this observation. These findings imply that lower expression level of *ANRIL* in the risk allele is associated with reduced expressions of G1 cell-cycle inhibitors, *p16*^*INK4A*^ and *p15*^*INK4B*^, which may contribute to a proliferative phenotype of ectopic endometrial cells and promote the development of endometriosis. To uncover modular regulatory mechanisms of the candidate SNP, Wnt signaling, and *ANRIL* expression, which leads to the development of endometriosis, further functional studies are required. A straightforward approach is to evaluate effect of disrupting the binding motif of HMG class of TFs at the SNP site on *ANRIL* and genome-wide expression profiles and cellular phenotypes, because it has been reported that *ANRIL* regulates gene expression network in a *trans*-acting manner [47, 48].

Genetic variants on 9p21 have been reported to be associated with numerous diseases such as CAD [49], intracranial aneurysm [50], type 2 diabetes [51], glaucoma [52], and several types of cancers [e.g., 53]. Harismendy et al. demonstrated that a 9p21 CAD associated SNP rs10757278 disrupted a binding site for STAT1, and this enhancer site modulated *ANRIL* expression via IFN-γ stimulation [15]. Interestingly, the binding of STAT1 at the enhancer site exerted cell-type specific regulations of *ANRIL* (repression in lymphoblastoid cells lines; activation in HUVEC) [15]. *ANRIL* is transcribed into several different splicing variants. The risk haplotype for CAD have been shown to be consistently associated with decreased levels of isoforms containing the 5’ exons [54], which is similar to our ASE result. The elucidation of tissue-specific regulatory mechanisms of *ANRIL* isoforms may clarify the pleiotropic effects of 9p21 region.

Limitations of our study should be noted. The methods of prioritizing two SNPs (rs17761446 and rs17834457) for subsequent functional studies were based on several criteria: degree of LD with the original GWAS SNPs; DNase-seq data from ENCODE cell types; and detection of significant DHSs. Although the inclusion criteria were set in order to elucidate more generalized functional aspect, we can not rule out a possibility that the variants that do not fulfill the criteria can be responsible for the 9p21 endometriosis risk locus. We prioritized rs17761446 over rs17834457 based on the motif search and the ENCODE ChIP-seq analyses. As both of these two analyses are incomplete, there is a possibility that uncharted TFs bind to rs17834457. It is also plausible that both rs17761446 and rs17834457 facilitate as a *cis*-regulatory haplotype in a coordinated fashion. In order to evaluate whether the haplotype constructed by these variants or single variant is truly functional, it is useful to create mutant cell lines that harboring these variants separately or some combinations by using genome editing technique such as CRISPR/Cas9 system [55, 56].

Among the SNPs identified by GWASs of endometriosis, most replicated SNP is located on chromosome 1p36 in a region close to *WNT4* [31–33]. Together with our results, these findings raise the possibility that Wnt signaling pathway is involved in the pathogenesis of endometriosis. The regulatory mechanisms of these susceptibility loci might converge to shared molecular network underlying the pathogenesis of endometriosis, which will provide clues for potential targets exploited for new drug development.

## Materials and Methods

### Study subjects

Study subjects were Japanese and recruited at Niigata University and Nippon Medical School. All the participants provided written informed consent. The Ethics Committee of National Institute of Genetics (nig1128, 2012. 10. 9), Niigata University (488, 2014. 1. 27), and Nippon Medical School (24-12-273, 2014. 3. 15) approved the study protocols.

### Target re-sequencing of 9p21 region

We selected 1.29 Mb interval of chromosome 9 (chr9:21299764–22590271, hg19) as a target region for next-generation sequencing (S1 Fig). The method for selecting target region is described in S1 Fig. We used NimbleGen SeqCap EZ choice system as a target enrichment method (Roche Diagnostics). A DNA probe set complementary to the target region was designed by NimbleDesign (https://design.nimblegen.com).

Genomic DNA was prepared from 48 Japanese patients with endometriosis using QIAamp DNA Blood Maxi Kit (QIAGEN) according to the manufacturer’s protocol as previously described [30, 57]. For each sample, 1 μg of genomic DNA was sheared into fragments with a mode length of about 200 bp on the Covaris (Covaris). Sequencing libraries were constructed with the Illumina TruSeq DNA sample Preparation Kit with 12 different indexed adapters (Illumina). Then, the target enrichment was performed with NimbleGen SeqCap EZ choice system according to the standard protocol (Roche Diagnostics). The libraries were sequenced on four runs of the Illumina MiSeq platform with 2×150-bp paired-end module (Illumina).

The reads containing the Illumina adapter sequences were trimmed by using Trimmomatic version 0.32 [58]. After the quality control step for excluding or trimming low quality sequences, the sequence reads were aligned to human reference genome (hg19) via BWA version 0.7 [59]. The aligned reads were processed for removal of PCR duplicates and erroneous reads by Picard tools version 1.111, and for local realignment and base quality recalibration by GATK version 3.2.2 [60, 61]. SNVs and indels were detected with the HaplotypeCaller and VariantRecalibrator of the GATK version 3.2.2 [60, 61].

### DNase-seq data from the ENCODE project

We analyzed DNase-seq data generated by Duke University in the ENCODE consortium to explore the distribution of DHSs. The sequence alignment files in the BAM format were downloaded from the ENCODE public download repository at http://genome.ucsc.edu/ENCODE/downloads.html. The peak call for the identification of regions in which aligned reads were significantly enriched was implemented via F-Seq version 1.84 with the use of background model for 20 bp sequences [62].

### Cell lines

Endometrial carcinoma cell lines (HEC251 [JCRB1141] and HEC265 [JCRB1142]) were grown in DMEM (Sigma-Aldrich) with 10% FBS. These cell lines were purchased from JCRB Cell Bank. We confirmed that HEC251 and HEC265 were heterozygous for rs17761446 by Sanger sequencing of the PCR product with the primer pair listed in S3 Table.

### 3C assay

The 3C libraries from HEC251 and HEC265 were generated with the established protocol (S10A Fig) [34, 63]. HEC251 and HEC265 cells (1×10^8^ cells per experiment) were crosslinked with 1% formaldehyde for 12 min at room temperature followed by adding glycine to a final concentration of 0.125M to stop further crosslinking. The crosslinked cells were lysed with the buffer (10 mM Tris-HCl, pH = 8.0, 10 mM NaCl, 0.2% IGEPAL CA-630 Protease Inhibitor (EDTA free)) using the Dounce homogenizer. The cell pellets were treated with corresponding buffers to restriction enzymes and 1% SDS, and then incubated at 65°C for 10 min to remove non-crosslinked proteins from the DNA. Placed on ice, 10% Triton X-100 was added to quench the SDS. For digestion, we used “6 bp-cutter” restriction enzymes (*Eco*RI or *Hin*dIII) followed by incubation at 37°C overnight with rotation. T4 DNA ligase was used to create intra-molecule ligations. The crosslinks were degraded by proteinase K treatment at 65°C overnight followed by an additional 2 h incubation at 42°C. The 3C DNA was purified by two rounds of phenol-chloroform (pH = 7.9) method followed by five ethanol washes. The unidirectional primer design [63] was used for examining chromatin loops formed between the restriction fragment containing candidate causal SNPs and the consecutive restriction fragments around *ANRIL*, *CDKN2A*, and *CDKN2B* (S10B Fig). The designed primers are shown in S2 Table.

### AS3C-seq

The workflow of the AS3C is illustrated in S10 Fig. We used HEC251 and HEC265 cells that were heterozygous for rs17761446. By using *Hin*dIII, we could design the primers whose product contained the ligation junction and the SNP site at tractable size (1,225 bp; S11 Fig and S2 Table). The PCR product was gel-purified and then subjected to transposase-based library construction with the Nextera DNA Sample Preparation Kit (Illumina), which allowed simultaneous DNA fragmentation and adaptor ligation. The DNA libraries were pooled according to their molar concentrations evaluated by the Agilent High Sensitivity DNA Kit and 2100 Bioanalyzer (Agilent Technologies). The DNA libraries were sequenced on the MiSeq platform with 2×150-bp paired-end module (Illumina).

The bioinformatics analysis in AS3C-seq is described in S10C Fig. For the sequence reads aligned to “artificial chromosome” that was an exact sequence of the 3C PCR product, the number of paired-end read tag counts supporting reference and alternative alleles was measured. The allelic imbalance between two alleles of the SNP in the AS3C-seq reads was evaluated by assuming that allele-specific paired-end read tag counts follows a binomial distribution. For each experiment, the deviation from balanced allele-specific paired-end read tag counts (50:50) was examined by the binomial test (two-sided). When results from multiple experiments were combined, the likelihood ratio test was examined as follows:

$$LRT=2\text{log}\frac{L\left(\pi_{1}\right)}{L\left(\pi_{0}\right)}=2\sum_{i}[n_{iA}\text{log}\frac{\pi_{1}}{\pi_{0}}+\left(n_{i}-n_{iA}\right)\text{log}\frac{1-\pi_{1}}{1-\pi_{0}}],$$

where *LRT* statistic follows a chi-square distribution with degrees of freedom of 1, *π*_0_ and *π*_1_ are the proportion of allele-specific reads supporting “A” allele for the SNP site under the null and alternative hypotheses, respectively. For *i*th experiment, *n*_*i*_ is the total number of reads mapped to the SNP site and *n*_*iA*_ is the number of reads supporting “A” allele. Under the null hypothesis, *π*_0_ is set to be 0.5. *π*_1_ is defined as a weighted proportion across experiments: $\pi_{1}=\sum_{i}n_{iA}/\sum_{i}n_{i}$.

### *In silico* TF binding site search

61-nt sequences of which candidate SNP sites were located in the middle were retrieved from hg19. Two sequences with reference or alternative allele were generated for each candidate SNP. The PWMs were constructed based on the HOCOMOCO database [36]. MotifSuite was used to detect motifs within the query sequences showing better fits to the HOCOMOCO PWMs over the background model for human genome [64].

### ChIP-seq data from the ENCODE project

ChIP-seq data in HeLa-S3 cells were downloaded from the ENCODE public download repository at http://genome.ucsc.edu/ENCODE/downloads. Significant peaks for the bindings of TCF7L2, EP300, and TBP were determined based on the ENCODE narrow peak regions. The enrichment of a histone modification, H3K27ac, was defined as the ENCODE broad peak regions.

### ChIP assay

ChIP assays for HEC251 cells were performed with SimpleChIP Plus Enzymatic Chromatin IP Kit (Cell Signaling) according to the manufacturer’s protocol. Briefly, cells were crosslinked with 1% formaldehyde for 10 min at room temperature, then treated with micrococcal nuclease to obtain fragments with a length of approximately 150–900 bp. The fragmented chromatin was subjected to immunoprecipitation with antibodies against TCF7L2, EP300 RNA polymerase II, H3K27ac, and SOX4. Normal rabbit IgG was used as negative control. The purified immunoprecipitated chromatin, input chromatin, and mock-IP (IgG) was subjected to PCR amplification of the candidate SNP site by using oligonucleotides primers (S3 Table). The binding affinities were measured by real-time quantitative PCR with the KAPA SYBR FAST qPCR kit (KAPA Biosystems) on the 7900HT sequence detection system (Applied Biosystems). The binding affinity in the immunoprecipitated chromatin was normalized to that in the corresponding input chromatin as follows: $2^{-\left(Ct_{IP}-Ct_{Input}\right)}$. The enrichment of the normalized binding affinity for the candidate SNP site or positive control region was normalized by dividing by that for negative control region. We used *MYC* promoter and α satellite repeat element as positive and negative control regions, respectively (S3 Table). Details on the antibodies are shown in S4 Table.

### Allele-specific factor binding analysis

The allele-specific TF binding at rs17761446 was determined using the TaqMan-based allelic discrimination assay. We used TaqMan SNP genotyping assay for rs17761446 (Applied Biosystems, Assay ID: C__33349228_10). In order to measure allelic ratios for the immunoprecipitated and input chromatins from HEC251 cells, a standard curve of the VIC/FAM ratios for the samples with known genotypes was generated as follows. Calibration of the allelic discrimination assay was determined by mixing three pairs of DNA samples with GG and TT homozygous genotypes for rs17761446 at the following proportions: 50:50, 60:40, 70:30, 80:20, and 90:10. DNA samples with GG, GT, and TT genotypes were also analyzed in the calibration assay. The standard curve was constructed by regressing the log2 transformed ratios for the VIC/FAM intensity on the log 2 transformed ratios of the two alleles. The allelic imbalance was examined by comparing the VIC/FAM ratios between the immunoprecipitated and input chromatins with the paired *t*-test (two-sided).

### ASE analysis

We performed the ASE analyses of rs17761446 for *ANRIL*, *CDKN2A*, and *CDKN2B* by using eutopic endometrial tissues and endometrial carcinoma cell lines. The eutopic endometrial tissues were provided by patients with ovarian cysts who underwent a total hysterectomy. All the specimens were snap-frozen in liquid nitrogen and stored at −80°C. Six patients with ovarian cysts and two endometrial carcinoma cell lines (HEC251 and HEC265) that were heterozygous for rs17761446 were used for further analyses. We performed target re-sequencing of the 9p21 region with the same methods described in the previous section. The haplotype phasing for the genotype data of these 8 samples along with the 48 samples in the previous section was implemented via Beagle 4.0 [65]. We found that the five patients with ovarian cysts and two endometrial carcinoma cell lines harbored heterozygous genotypes both for rs17761446 and a SNP within *ANRIL* (rs10965215) with the same haplotypes. In total, six and seven samples were available for the ASE analyses by using rs3814960 and rs3217992 as transcribed SNPs in *CDKN2A* and *CDKN2B*, respectively. LD structure for these SNPs are shown in S23 Fig.

RNA was extracted from eutopic endometrial tissues and endometrial carcinoma cell lines using AllPrep DNA/RNA Mini kit (QIAGEN) according to the manufacturer's instructions. 1 μg of the total RNA was reverse transcribed by using ReverTra Ace-α kit (Toyobo) with random primer. The synthesized cDNA was subject to PCR amplification by using oligonucleotide primers for the three 9p21 genes (S3 Table). The genomic DNA was also subjected to PCR amplification by using oligonucleotides primers (S3 Table), and used as control. The obtained PCR products were converted into indexed libraries by using NEBNext Ultra DNA Library Prep Kit for Illumina (New England Biolabs) followed by sequencing on the MiSeq platform with 250-bp paired-end module (Illumina). The generated reads were aligned to “artificial chromosomes”, which were exact sequences of the PCR products. The reads mapped with a high confidence (MAQ > 30) were used for the ASE analyses. The allele-specific paired-end tag counts were measured by using only high confidence base calls (base quality > 20) at the transcribed SNP positions. The allelic imbalance was examined by comparing the log 2 transformed ratios of the allele-specific paired-end tag counts between cDNA and genomic DNA with the paired *t*-test (two-sided).

### Change in expression levels of 9p21 genes by induction of Wnt signaling

CHIR (Tocris Bioscience) was dissolved in DMSO (Sigma). HEC251 cells were treated for 24 h with either DMSO (vehicle) or CHIR (5 μM), and then harvested for gene expression analysis. RNA was extracted from DMSO- and CHIR-treated HEC251 cells using AllPrep DNA/RNA Mini kit (QIAGEN) according to the manufacturer's instructions. 1 μg of the total RNA was reverse transcribed by using ReverTra Ace-αkit (Toyobo) with random primer. The expression levels were measured by real-time quantitative PCR with the KAPA SYBR FAST qPCR kit (KAPA Biosystems) on the 7900HT sequence detection system (Applied Biosystems). Three 9p21 genes (*ANRIL*, *CDKN2A*, and *CDKN2B*) were evaluated. Expression was normalized to *ACTB*. The oligonucleotides primers used are shown in S3 Table. The fold change was calculated by dividing expression level in CHIR-treated cells by that in cells treated with vehicle only (DMSO). Six replicates were performed. Difference in gene expression level between the two treatments was examined by testing whether log2 transformed fold change was different from zero with one sample *t*-test (two-sided).

### Immunofluorescence analysis

Immunofluorescence analysis was performed with CHIR- and DMSO-treated HEC251 cells. Cells grown on microscope slides were rinsed in Dulbecco’s PBS and then fixed in Bouin’s fixative (Sigma-Aldrich) for 5 min. The cells were incubated with the primary antibody against polyclonal β-catenin (S4 Table) over-night in a humidified chamber at 4°C. After washing, the cells were incubated with the secondary antibody (Alexa-Flour 488 goat anti-rabbit IgG; Life Technologies) in a humidified chamber for 1 h at room temperature. Finally, the cells were mounted with Vetashield HardSet Mounting Medium supplemented with DAPI (Vector Laboratories). Images acquisition was performed using Olympus FluoView^™^ FV1200 (Olympus).

### Assessment of mapping bias on allele specific functional genomic analyses

One reservation on allele specific functional genomics studies based on next generation sequencing technologies is mapping bias, in which reads with reference allele are more likely to be mapped than reads with alternative allele (i.e., bias toward preferential mapping to reference allele) [35]. Therefore, we addressed mapping bias on our AS3C-seq and ASE analyses by performing simulation and sensitivity analyses described as below.

(i) Simulation analysis

We performed a simulation analysis for evaluating mapping bias by patterning after three studies [35, 66, 67]. For AS3C-seq, we generated single-end reads based on the human reference genome (hg19). For all possible segments overlapping rs17761446 or rs17834457 at a defined sequence length, we generated a set of reads containing reference and alternative alleles. We considered random sequencing errors in the simulation, in which each base in the reads was substituted to a different randomly selected base with a Bernoulli probability of 0.001, 0.005, and 0.01. The error rate was determined based on Loman et al. reporting that MiSeq produced 0.1 mismatches per 100 bases [68]. We considered three simulation scenarios of different sequence lengths (50, 75, and 100 bp). We arbitrarily assigned the best base quality score of 41 to each base of the simulated reads.

rs17761446 and rs17834457 are closely located (76 bp apart) and in perfect LD (*r*^2^ = 1.0). We incorporated this fact into our simulation. When the sequence length was 100 bp, some of the sequence segments overlapped both of these SNPs. In such case, the haplotype structure observed in Japanese population was reflected in the simulation: reference T allele at rs17761446 resided on the same haplotype with reference C allele at rs17834457.

The simulated reads were mapped to the human reference genome rather than the artificial chromosome. The SNP sites were distant from a ligation junction between fragments containing the SNPs and *ANRIL* promoter (>700 bp), indicating that any single reads overlapping the SNPs did not reach to the ligation junction. Therefore, mapping to the reference genome are thought to be sufficient in order to evaluate effects of mapping bias at the SNP sites. BWA with default setting was used. For the reads with mapping quality > 30, we measured allele-specific read counts at the SNP sites and calculated proportion of the reads supporting reference allele. For each parameter combination, 10,000 simulations were carried out. For allele specific expression analyses, we conducted simulations by using the sequences of transcripts rather than genome.

The results of the simulations for allele specific chromatin interaction and allele specific expression analyses are shown in Figs 2D and 4C, respectively. The results show that when the sequence length is shorter and the error rate is higher, degree of preferential mapping to reference allele increases. The averaged proportion of mapped reads supporting reference allele did not surpass 52%, even when the error rate is set to be 0.01 and the sequence length is 50 bp, in which the error rate is ten times as large as a literature value for Illumina platform [68]. When we consider more realistic scenarios (i.e., the error rate of 0.001 and 0.005), the average proportion of mapped reads supporting reference allele was at most 50.03% and 50.06%, respectively. The results of the simulation showed very subtle preferences toward reference alleles, indicating that the mapping bias expected by the simulations does not affect our results.

(ii) Sensitivity analysis

In order to evaluate effects of mapping bias not covered by the simulation study, we performed a sensitivity analysis in which a base at SNP site in the reference sequence is changed to alternative allele. If a SNP site is susceptible to mapping bias, change from reference to alternative allele may diminish a preference to reference allele and exaggerate a preference to alternative allele. We aligned the reads generated by our allele specific chromatin interaction and allele specific expression analyses to the “original” and “changed” reference sequences with the same method. Then, differences in allelic imbalance were examined between two reference sequences.

The results of the sensitivity analyses for allele specific chromatin interaction and allele specific expression analyses are shown in Figs 2E and 4D, respectively. For the SNP sites analyzed, differences in allelic imbalance were small between two reference sequences (at most 0.15%).

According to the two analyses, we confirmed that mapping bias does not affect our results. Additionally, both of the significant results from our AS3C-seq and ASE exhibited increased proportions of alternative alleles. Therefore, it is most likely that the observed allelic imbalances in our study were not caused by mapping bias.

### Data access

Sequence data for re-sequencing of 9p21 region has been deposited at the European Genome-phenome Archive (EGA), which is hosted by the EBI and the CRG, under accession number EGAS00001001741/EGAD00001001942. Nucleotide sequence data for AS3C-seq are available in the DDBJ Sequenced Read Archive under the accession numbers DRX051835 and DRX051836.

## Supporting Information

S1 Fig 1.29 Mb interval on chromosome 9p21 selected for target re-sequencing. (PDF)

S2 Fig Coverages of target and baited regions for 48 samples according to the date of MiSeq run. (PDF)

S3 Fig Averages of depth over target and baited regions for 48 samples according to the date of MiSeq run. (PDF)

S4 Fig Transition/transversion ratio for 9p21 target region in the samples from current study and the 1000 Genomes project. (PDF)

S5 Fig Characteristics of detected 4,215 SNVs and 664 indels. (PDF)

S6 Fig Frequency distributions of detected SNVs and indels. (PDF)

S7 Fig Extent of linkage disequilibrium (*r*^2^) between each pair of two endometriosis-associated SNPs (rs10965235 and rs1537377) and GWAS tag SNPs on 9p21 associated with other diseases. (PDF)

S8 Fig Distributions of DHSs in representative ENCODE cell lines and endometrial carcinoma cell lines. (PDF)

S9 Fig PCR amplification of the interacting fragments between candidate SNPs and the promoter of *ANRIL*. A) Consistent PCR amplifications for the detection of chromatin interaction between the fragment containing candidate SNPs (rs17761446 and rs17834457) and the fragment containing the promoter of ANRIL in HEC251 and HEC265 cell lines. B) Sanger sequence for the PCR amplicon for the interacting fragments between candidate SNPs (rs17761446 and rs17834457) and the promoter of *ANRIL* verified the presence of the ligation junction of these two fragments.
(PDF)

S10 Fig Workflow of AS3C-seq. A) 3C library preparation. B) PCR amplification followed by high-throughput sequencing. C) Bioinformatics analysis.
(PDF)

S11 Fig Unidirectional primer design for the detection of chromatin interaction between fragment containing SNP rs17761446 and fragment containing the transcription start site (TSS) of *ANRIL*. (PDF)

S12 Fig Homopolymer stretch at the site with lower coverage of depth in AS3C-seq data. (PDF)

S13 Fig Transcription factor binding motif search around rs17761446. (PDF)

S14 Fig Transcription factor binding motif search around rs17834457. (PDF)

S15 Fig Alignments of consensus motifs of SOX family to sequence surrounding rs17761446. (PDF)

S16 Fig Standard curve of the VIC/FAM ratios by TaqMan-based allelic discrimination assay. (PDF)

S17 Fig Quantification of allele-specific factor bindings at rs17761446 based on the standard curve. A) VIC/FAM ratios for immunoprecipitated and input chromatins by TaqMan allelic discrimination assay. B) Allelic ratios for ChIP libraries determined based on the standard curve.
(PDF)

S18 Fig Allele-specific factor bindings in HEC251 cells. (PDF)

S19 Fig Immunofluorescence analysis for CHIR- and DMSO-treated HEC251 cells. (PDF)

S20 Fig Induction of Wnt signaling alters expression levels of 9p21 genes. A) Structures of ANRIL transcripts amplified by three primer pairs. B) Fold expressions of CHIR-treated cells for transcripts of *ANRIL*, *CDKN2A* and *CDKN2B* relative to cells treated with vehicle only (DMSO).
(PDF)

S21 Fig ASE analyses of rs17761446 for *ANRIL* in CHIR- and DMSO-treated HEC251 cells. (PDF)

S22 Fig ChIP assays for SOX4 at rs17761446 site in HEC251 cells. (PDF)

S23 Fig LD structure for SNPs used in ASE analyses. (PDF)

S1 Table Variants exhibiting strong LD with two SNPs identified by the original GWASs. (PDF)

S2 Table Oligonucleotides used in 3C. (PDF)

S3 Table PCR primers for SNP genotyping, ChIP assay and gene expression analysis. (PDF)

S4 Table Antibodies used in ChIP and immunofluorescence analyses. (PDF)
