# Supplementary material for: Allelic Imbalance in Regulation of ANRIL through Chromatin Interaction at 9p21 Endometriosis Risk Locus
Source: PLoS Genet. 2016 Apr 7;12(4):e1005893. doi: 10.1371/journal.pgen.1005893 (PMC4824487; doi:10.1371/journal.pgen.1005893)
Supplement: S1 Fig — (PDF) [file pgen.1005893.s001.pdf]

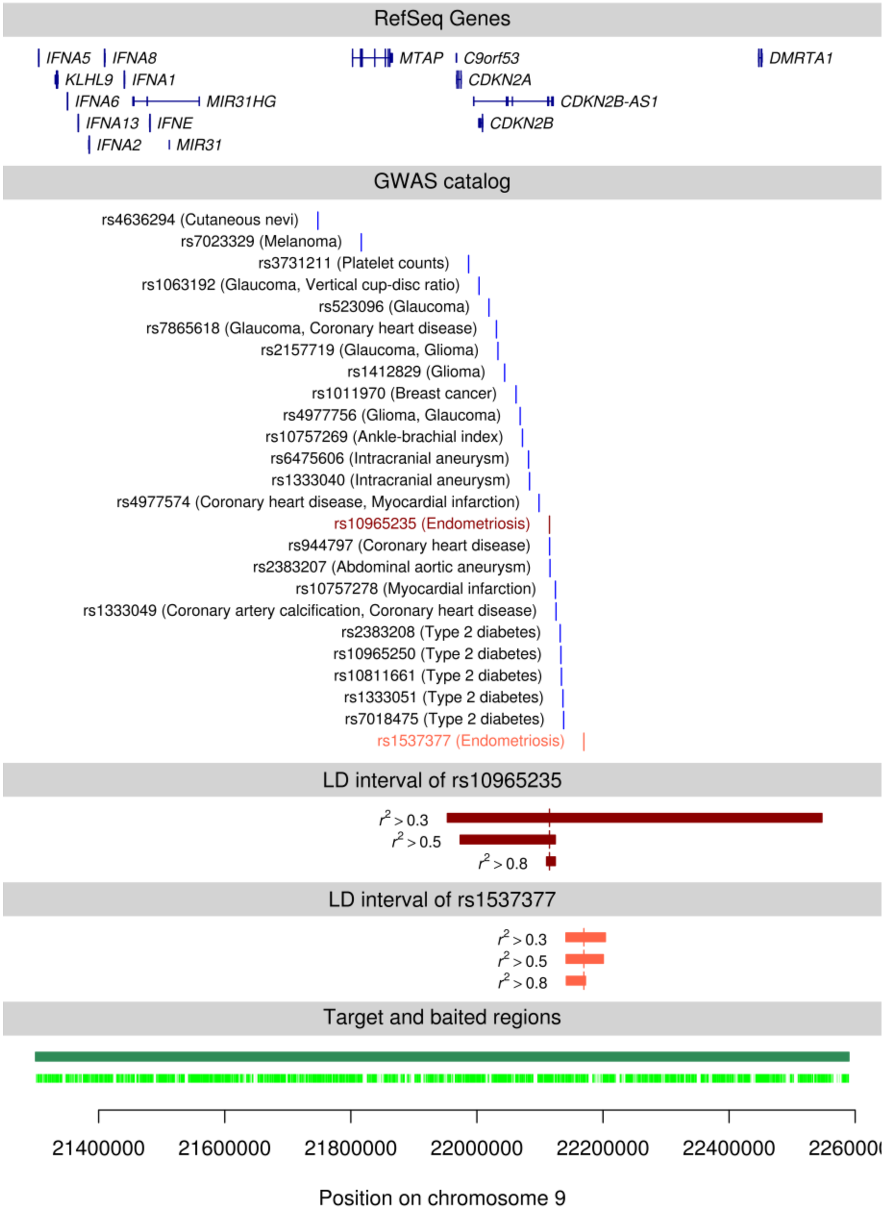

S1 Fig. 1.29 Mb interval on chromosome 9p21 selected for target re-sequencing. The target region (chr9: 21299764-22590271, hg19 [green rectangle in bottom track]) was selected as follows: i) it encompassed 400 kb region across any of the disease-associated SNPs on 9p21.3 satisfying the conventional genome-wide significant threshold (P<5.0×10^{-8}) in NHGRI GWAS catalog (second track); and ii) interval contained all the HapMap SNPs that were in LD with two endometriosis-associated SNPs (rs10965235 [third track] and rs1537377 [fourth track]). The DNA probe set complementary to the target region is shown as baited region (light green tiles in bottom track). RefSeq Genes on the corresponding region are in top track. The tiled probe set covers 77.5% of the target bases (baited region). Given that regions within 100 bp flanking both sides of each bait can be captured, 87.2% of the target bases is estimated to be covered.

The target region was selected as follows: The interval contains all the SNPs that are in LD with rs10965235 and rs1537377 at $r^2 > 0.3$ in JPT samples from HapMap phase II+III data set (International HapMap 3 Consortium et al., 2010). Furthermore, the target region encompasses 400 kb region across any of the disease-associated SNPs on 9p21.3 region surpassing the conventional genome-wide significance threshold ($P < 5.0 \times 10^{-8}$) in the NHGRI GWAS catalog at the time of deposition of Nyholt et al. (January of 2013) (Welter et al., 2014).

International HapMap 3 Consortium, et al. Integrating common and rare genetic variation in diverse human populations. *Nature*. 2010; 467(7311): 52-58.
Welter D, et al. The NHGRI GWAS Catalog, a curated resource of SNP-trait associations. *Nucleic Acids Res*. 2014; 42(Database issue): D1001-D1006.
