# Supplementary material for: Allelic Imbalance in Regulation of ANRIL through Chromatin Interaction at 9p21 Endometriosis Risk Locus
Source: PLoS Genet. 2016 Apr 7;12(4):e1005893. doi: 10.1371/journal.pgen.1005893 (PMC4824487; doi:10.1371/journal.pgen.1005893)
Supplement: S2 Fig — (PDF) [file pgen.1005893.s002.pdf]

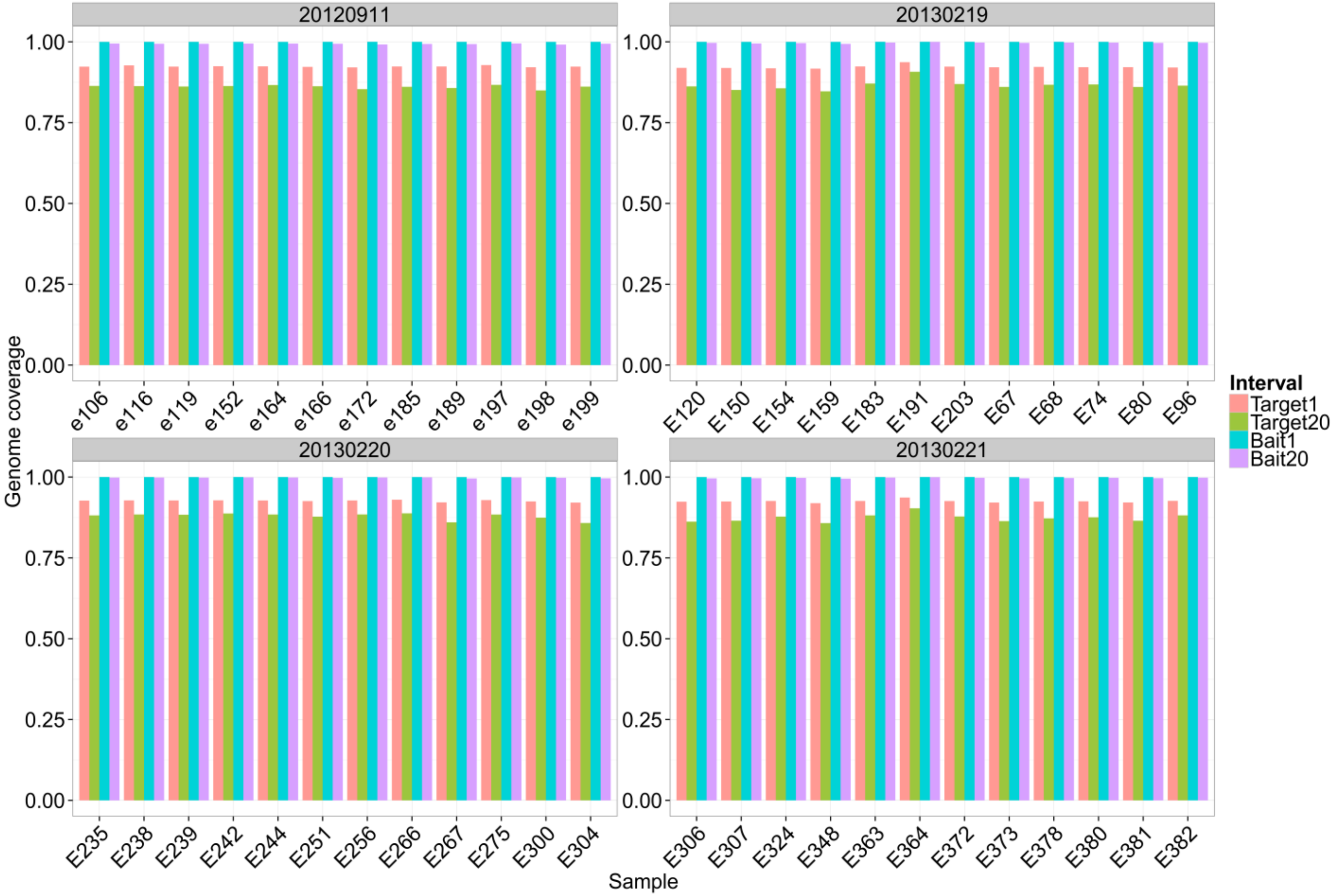

S2 Fig. Coverages of target and baited regions for 48 samples according to the date of MiSeq run.
Proportions of target or baited regions covered by at least 1 or 20 reads are shown.
