# Supplementary material for: Allelic Imbalance in Regulation of ANRIL through Chromatin Interaction at 9p21 Endometriosis Risk Locus
Source: PLoS Genet. 2016 Apr 7;12(4):e1005893. doi: 10.1371/journal.pgen.1005893 (PMC4824487; doi:10.1371/journal.pgen.1005893)
Supplement: S3 Fig — (PDF) [file pgen.1005893.s003.pdf]

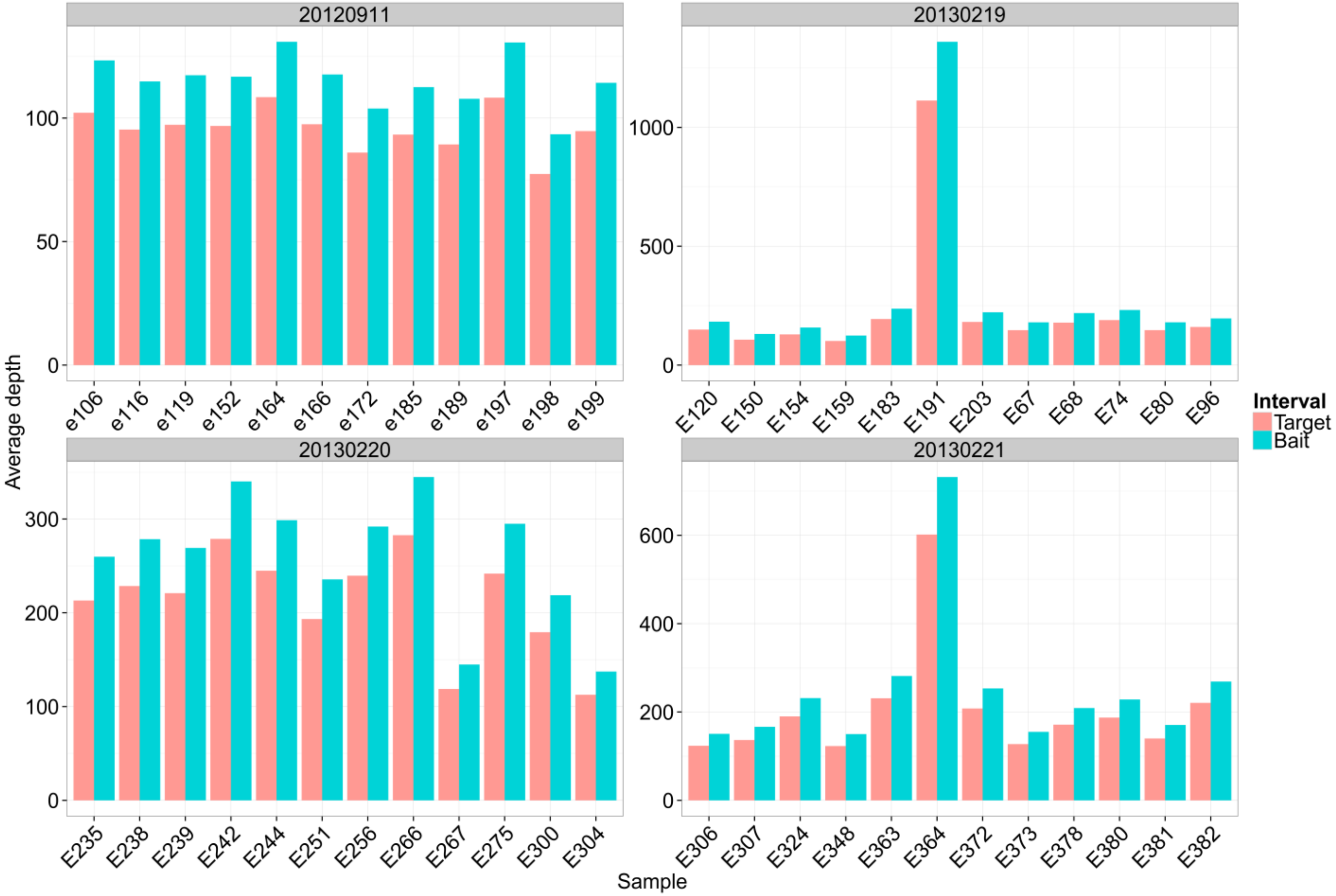

S3 Fig. Averages of depth over target and baited regions for 48 samples according to the date of MiSeq run.
