# Supplementary material for: Allelic Imbalance in Regulation of ANRIL through Chromatin Interaction at 9p21 Endometriosis Risk Locus
Source: PLoS Genet. 2016 Apr 7;12(4):e1005893. doi: 10.1371/journal.pgen.1005893 (PMC4824487; doi:10.1371/journal.pgen.1005893)
Supplement: S4 Fig — (PDF) [file pgen.1005893.s004.pdf]

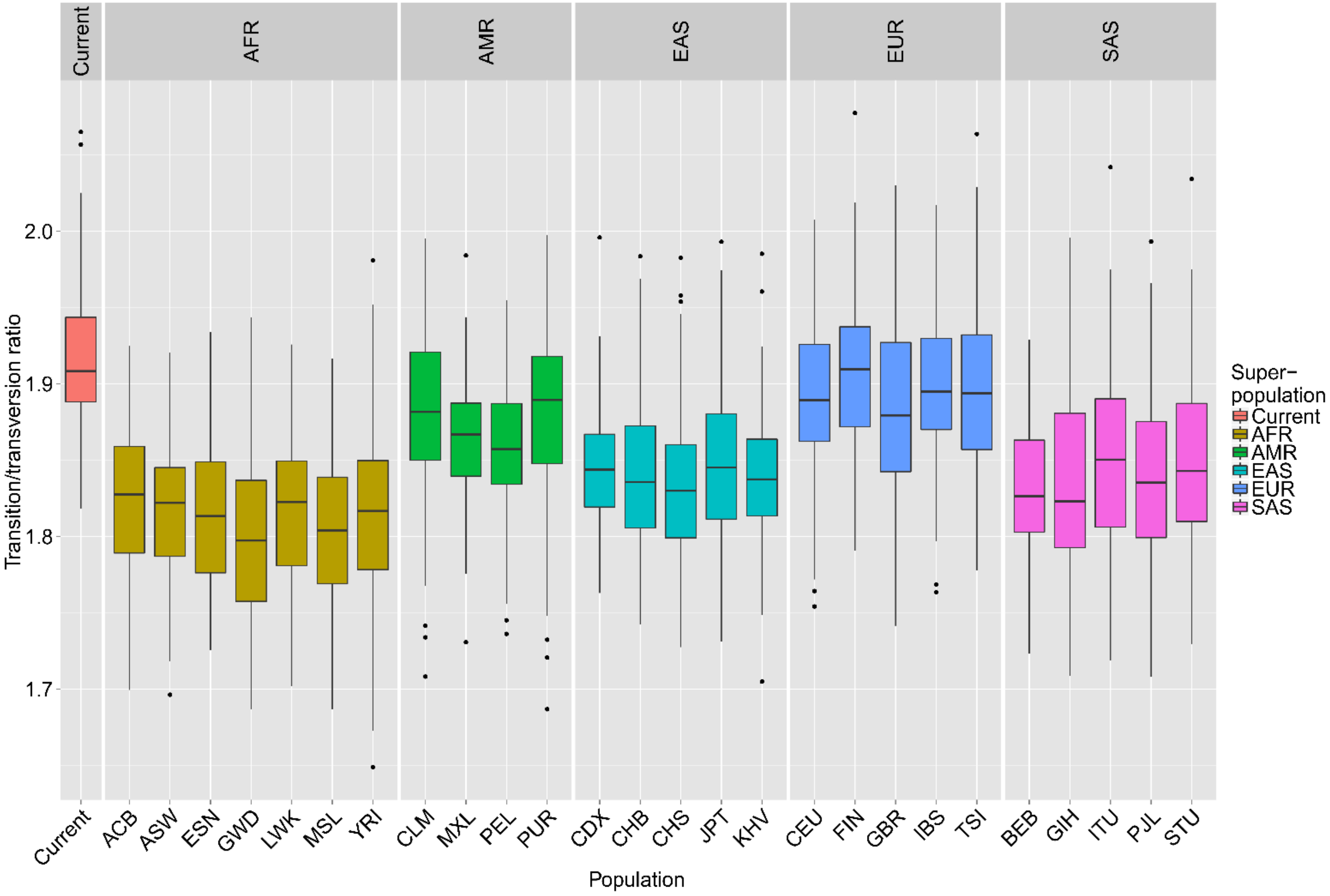

S4 Fig. Transition/transversion ratio for 9p21 target region in the samples from current study and the 1000 Genomes project. Twenty-six populations from the 1000 Genomes project are categorized into 5 super populations: AFR, African; AMR, Admixed American; EAS, East Asian; EUR, European; and SAS, South Asian.
