# Supplementary material for: Allelic Imbalance in Regulation of ANRIL through Chromatin Interaction at 9p21 Endometriosis Risk Locus
Source: PLoS Genet. 2016 Apr 7;12(4):e1005893. doi: 10.1371/journal.pgen.1005893 (PMC4824487; doi:10.1371/journal.pgen.1005893)
Supplement: S5 Fig — (PDF) [file pgen.1005893.s005.pdf]

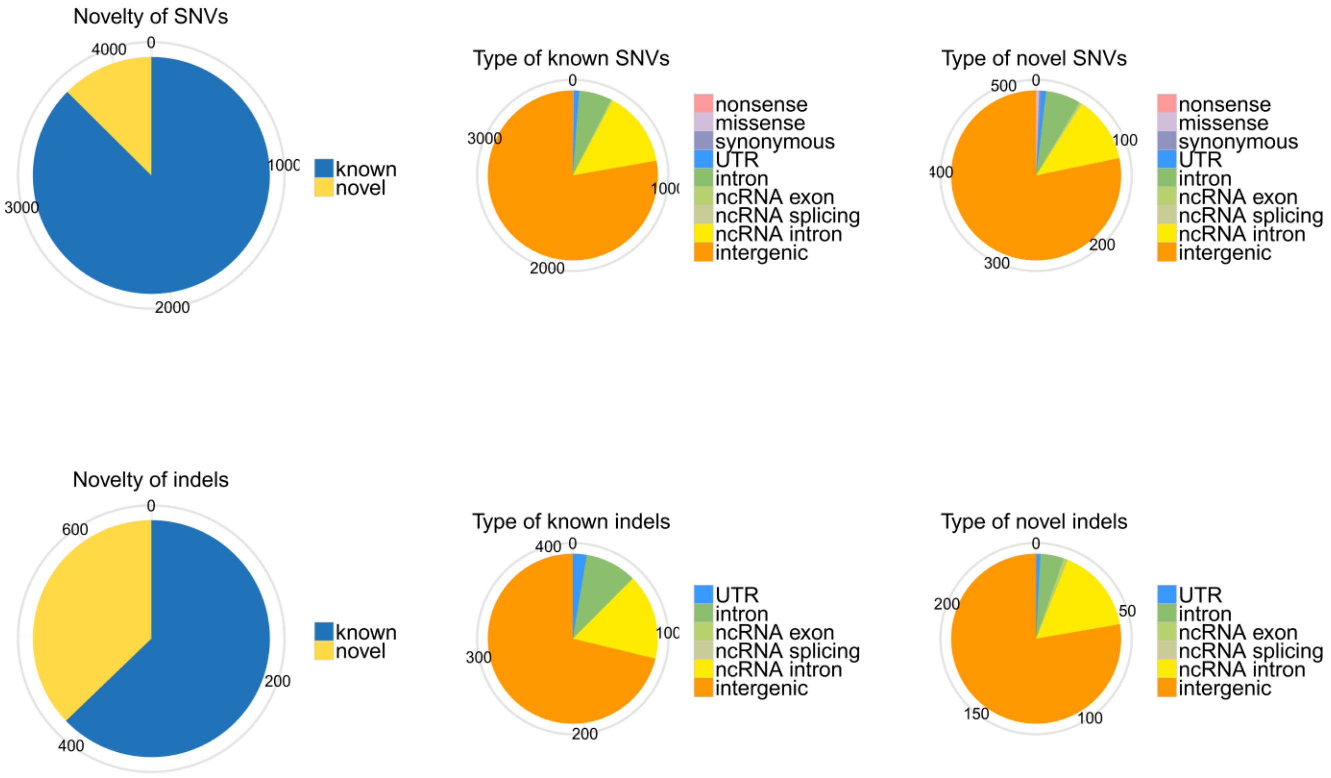

S5 Fig. Characteristics of detected 4,215 SNVs and 664 indels.

SNVs and indels were classified into known and novel variants according to the presence in NCBI dbSNP build 138. Functional annotation was implemented via ANNOVAR (Wang et al., 2010).

Wang K, Li M, Hakonarson H. ANNOVAR: functional annotation of genetic variants from high-throughput sequencing data. *Nucleic Acids Res*. 2010; 38(16): e164.
