# Supplementary material for: Allelic Imbalance in Regulation of ANRIL through Chromatin Interaction at 9p21 Endometriosis Risk Locus
Source: PLoS Genet. 2016 Apr 7;12(4):e1005893. doi: 10.1371/journal.pgen.1005893 (PMC4824487; doi:10.1371/journal.pgen.1005893)
Supplement: S6 Fig — (PDF) [file pgen.1005893.s006.pdf]

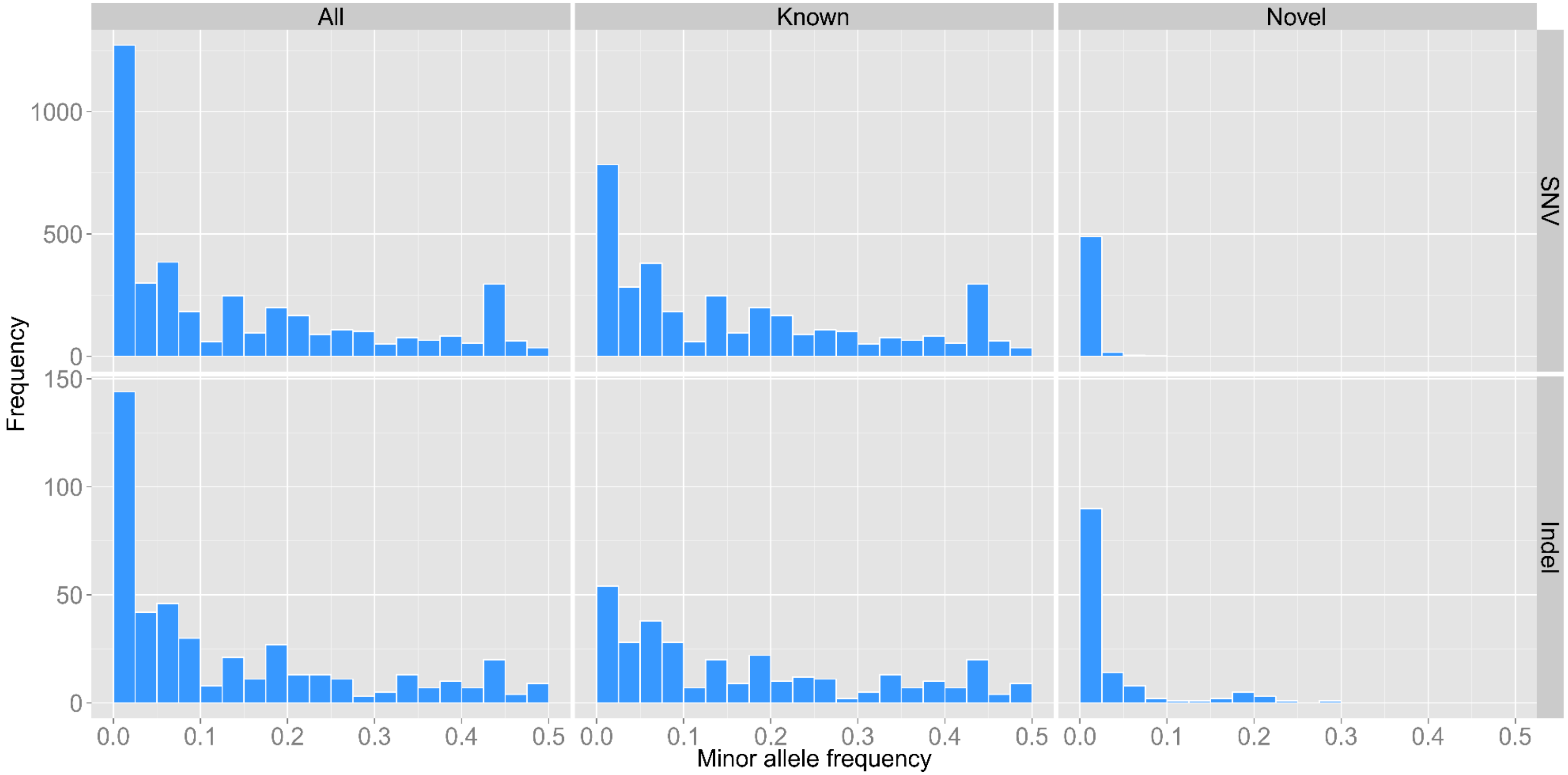

S6 Fig. Frequency distributions of detected SNVs and indels.

SNVs and indels were classified into known and novel variants according to the presence in NCBI dbSNP build 138. We excluded SNVs and indels whose missing genotype rates were greater than 0.10 and that were monomorphic (i.e., all the individuals were homozygous for the alternative allele).
