# Supplementary material for: Allelic Imbalance in Regulation of ANRIL through Chromatin Interaction at 9p21 Endometriosis Risk Locus
Source: PLoS Genet. 2016 Apr 7;12(4):e1005893. doi: 10.1371/journal.pgen.1005893 (PMC4824487; doi:10.1371/journal.pgen.1005893)
Supplement: S7 Fig — (PDF) [file pgen.1005893.s007.pdf]

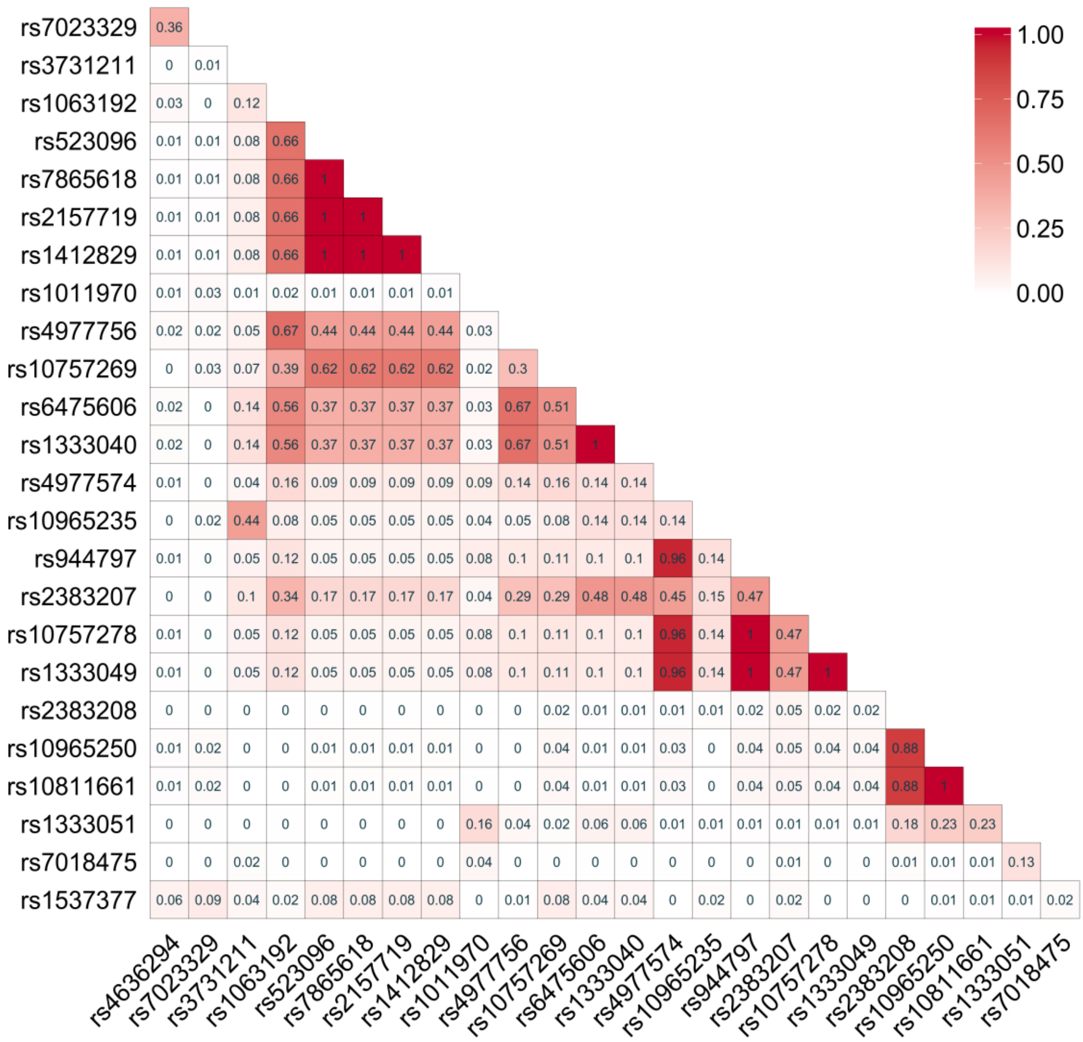

S7 Fig. Extent of linkage disequilibrium (r^2) between each pair of two endometriosis-associated SNPs (rs10965235 and rs1537377) and GWAS tag SNPs on 9p21 associated with other diseases.
GWAS tag SNPs were retrieved from NHGRI GWAS catalog, which are shown in Supplemental Figure 1. We evaluated extent of LD for each pair of rs10965235 or rs1537377 and the other variants by using PLINK v1.07 (Purcell et al., 2007).

Purcell S, et al. PLINK: a tool set for whole-genome association and population-based linkage analyses. *Am J Hum Genet*. 2007; 81(3): 559-575.
