# Supplementary material for: Allelic Imbalance in Regulation of ANRIL through Chromatin Interaction at 9p21 Endometriosis Risk Locus
Source: PLoS Genet. 2016 Apr 7;12(4):e1005893. doi: 10.1371/journal.pgen.1005893 (PMC4824487; doi:10.1371/journal.pgen.1005893)
Supplement: S8 Fig — (PDF) [file pgen.1005893.s008.pdf]

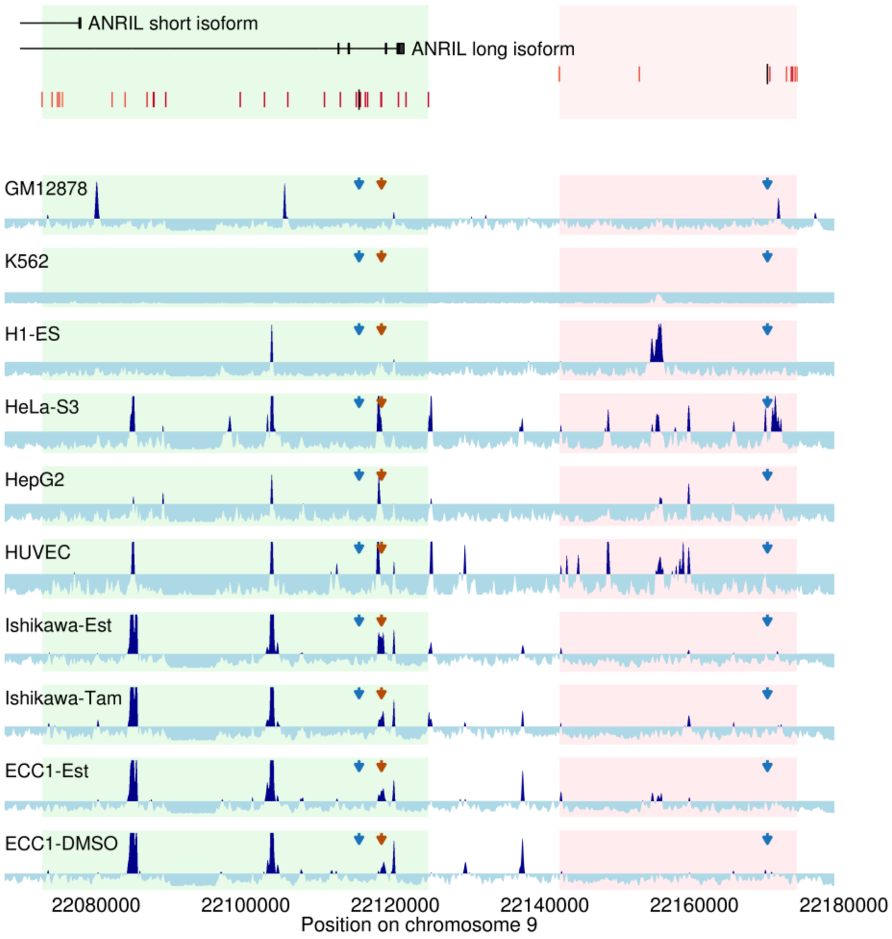

S8 Fig. Distributions of DHSs in representative ENCODE cell lines and endometrial carcinoma cell lines.

The intervals encompassing all the variants showing strong LD ($r^2 > 0.8$) with rs10965235 and rs1537377 are highlighted by green and pink shades, respectively. These intervals are located on 3' ends of *ANRIL* isoforms. The positions of the variants are depicted below transcript structures. The densities of aligned reads from DNase-seq estimated by F-Seq are plotted in the image of "iceberg". DHSs in which the densities of aligned reads significantly surpassing the threshold are represented as visible parts of an iceberg (dark blue). On the other hand, locations in which aligned reads are depleted are represented below the surface of the sea (light blue). The positions of the original GWAS SNPs (rs10965235 and rs1537377) and candidate causal variants (rs17761446 and rs17834457) are highlighted by blue and red arrows, respectively. The analyzed cell lines are as followed: GM12878 (B lymphoblastoid), K562 (chronic myeloid leukemia), H1-hESC (H1 embryonic stem cell), HeLa-S3 (cervical cancer), HepG2 (hepatocellular liver carcinoma), HUVEC (umbilical vein epithelial cell), and Ishikawa and ECC1 (endometrial carcinoma). Ishikawa cells were treated with estradiol (Ishikawa-Est) or tamoxifen (Ishikawa-Tam). ECC1 cells were treated with estradiol (ECC1-Est) or vehicle only (ECC1-DMSO).
