# Supplementary material for: Allelic Imbalance in Regulation of ANRIL through Chromatin Interaction at 9p21 Endometriosis Risk Locus
Source: PLoS Genet. 2016 Apr 7;12(4):e1005893. doi: 10.1371/journal.pgen.1005893 (PMC4824487; doi:10.1371/journal.pgen.1005893)
Supplement: S9 Fig — A) Consistent PCR amplifications for the detection of chromatin interaction between the fragment containing candidate SNPs (rs17761446 and rs17834457) and the fragment containing the promoter of ANRIL in HEC251 and HEC265 cell lines. B) Sanger sequence for the PCR amplicon for the interacting fragments between candidate SNPs (rs17761446 and rs17834457) and the promoter of ANRIL verified the presence of the ligation junction of these two fragments. (PDF) [file pgen.1005893.s009.pdf]

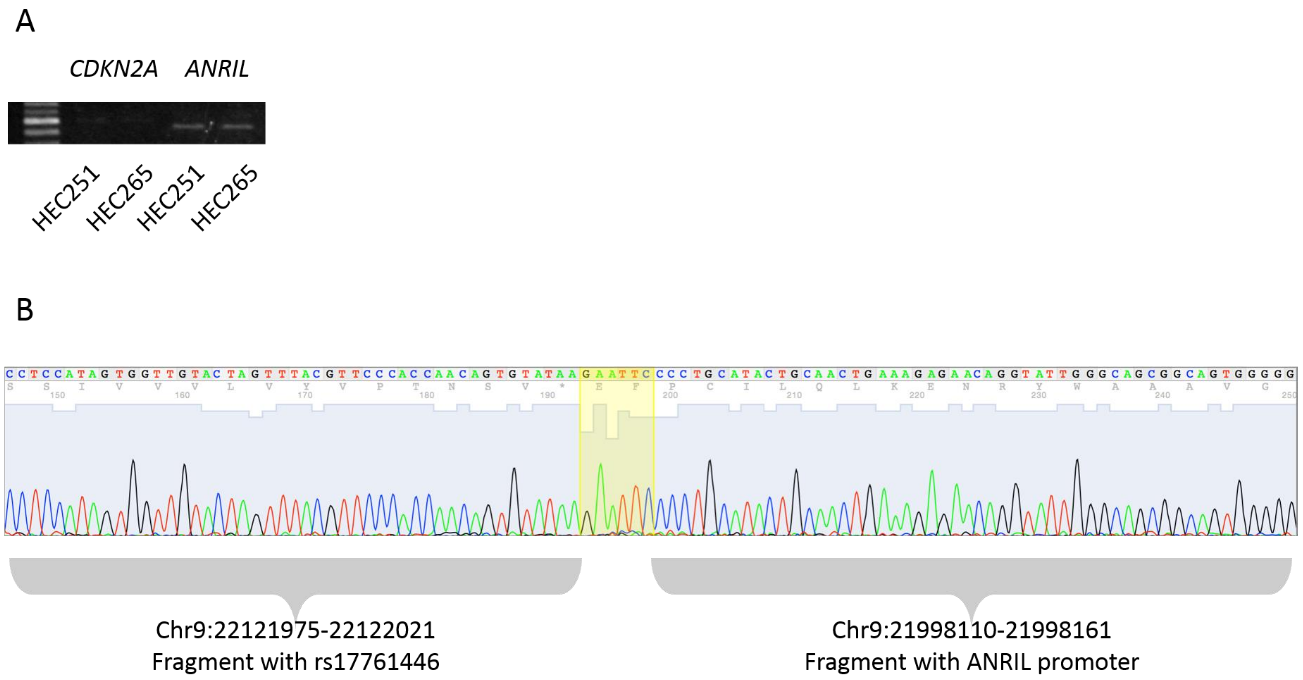

S9. PCR amplification of the interacting fragments between candidate SNPs and the promoter of *ANRIL*.

A) Consistent PCR amplifications for the detection of chromatin interaction between the fragment containing candidate SNPs (rs17761446 and rs17834457) and the fragment containing the promoter of *ANRIL* in HEC251 and HEC265 cell lines. The fragment containing rs17761446 shows much stronger interaction with the fragment containing the promoter of *ANRIL* than that with *CDKN2A* (background). 3C libraries are created by 6 bp-cutter restriction enzyme, *Eco*RI. Oligonucleotide primers are shown in S2 Table.

B) Sanger sequence for the PCR amplicon for the interacting fragments between candidate SNPs (rs17761446 and rs17834457) and the promoter of *ANRIL* verified the presence of the ligation junction of these two fragments. The sequences surrounding the *Eco*RI recognition motif (GAATTC; yellow) are matched to the sequences within the fragment containing candidate SNPs (rs17761446 and rs17834457) and the fragment containing the promoter of *ANRIL* by BLAT search on the UCSC Genome Browser.
