# Supplementary material for: Allelic Imbalance in Regulation of ANRIL through Chromatin Interaction at 9p21 Endometriosis Risk Locus
Source: PLoS Genet. 2016 Apr 7;12(4):e1005893. doi: 10.1371/journal.pgen.1005893 (PMC4824487; doi:10.1371/journal.pgen.1005893)
Supplement: S10 Fig — A) 3C library preparation. B) PCR amplification followed by high-throughput sequencing. C) Bioinformatics analysis. (PDF) [file pgen.1005893.s010.pdf]

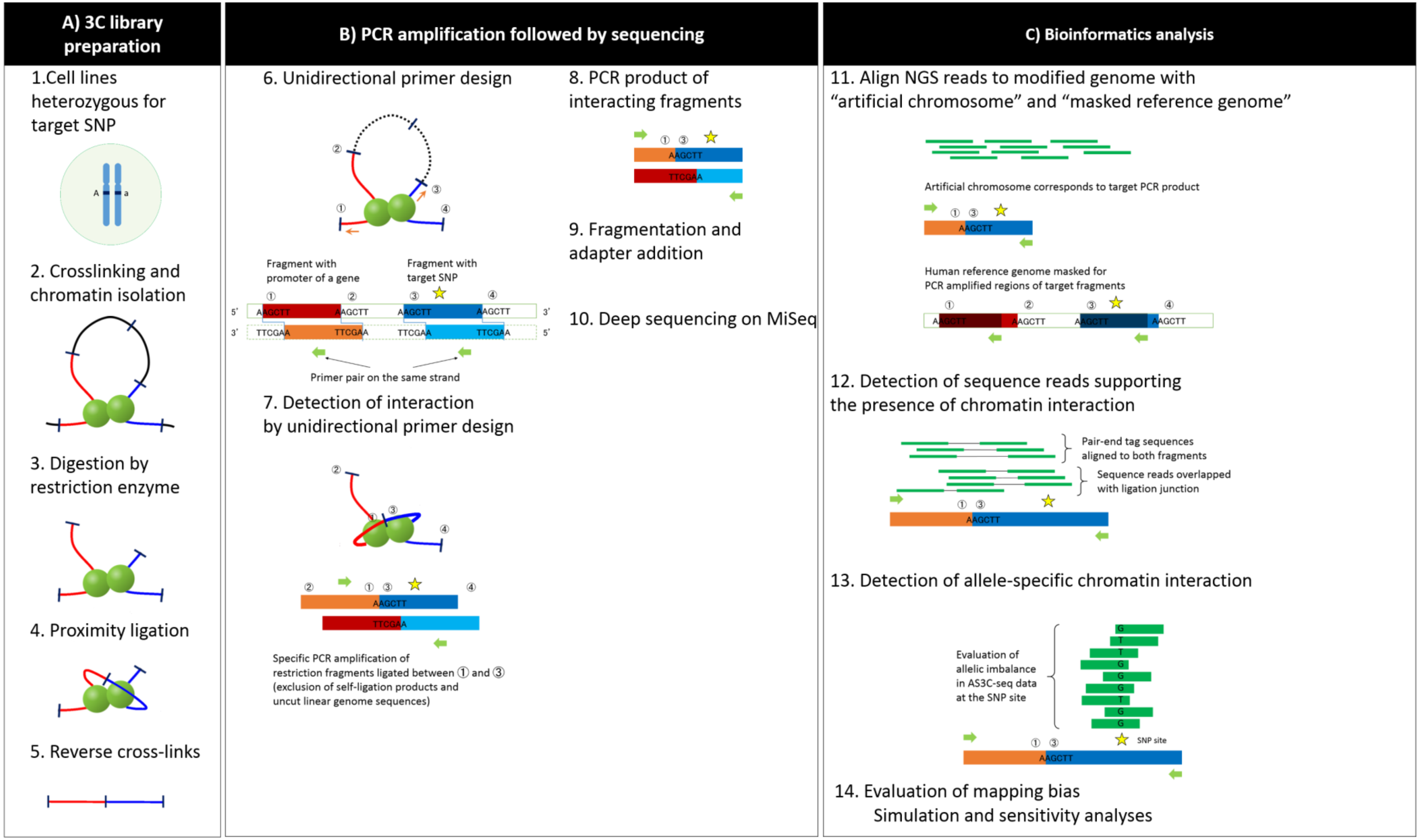
A) 3C library preparation
1.Cell lines heterozygous for target SNP
A
a
2. Crosslinking and chromatin isolation
3. Digestion by restriction enzyme
4. Proximity ligation
5. Reverse cross-links
B) PCR amplification followed by sequencing
6. Unidirectional primer design
Fragment with promoter of a gene
Fragment with target SNP
AAGCTT
TTCGAA
5'
3'
Primer pair on the same strand
7. Detection of interaction by unidirectional primer design
Specific PCR amplification of restriction fragments ligated between ① and ③ (exclusion of self-ligation products and uncut linear genome sequences)
8. PCR product of interacting fragments
9. Fragmentation and adapter addition
10. Deep sequencing on MiSeq
C) Bioinformatics analysis
11. Align NGS reads to modified genome with “artificial chromosome” and “masked reference genome”
Artificial chromosome corresponds to target PCR product
Human reference genome masked for PCR amplified regions of target fragments
12. Detection of sequence reads supporting the presence of chromatin interaction
Pair-end tag sequences aligned to both fragments
Sequence reads overlapped with ligation junction
13. Detection of allele-specific chromatin interaction
Evaluation of allelic imbalance in AS3C-seq data at the SNP site
SNP site
14. Evaluation of mapping bias
Simulation and sensitivity analyses

S10 Fig. Workflow of AS3C-seq.

A) The use of cell lines or tissues that are heterozygous for target SNP site is requisite. The 3C library preparation protocol involves cross-lining with formaldehyde, digestion with a restriction enzyme, intra-molecular ligation, and reverse cross-links.

B) The PCR amplification of ligation product with the unidirectional primer design followed by high-throughput DNA sequencing. The restriction enzyme recognition sites around a looping interaction between distant parts of the genome are numbered according to the genomic coordinates. The ligation products for any pair of the digested sites including self-ligations can be obtained. The unidirectional primer design specifically detects only one of the ligation products: if both of the primers are designed to bind to “forward strand”, only the product with the ligation junction between “2” and “4” is amplified. If they are designed to bind to “reverse strand”, only the product with the ligation junction between “1” and “3” is amplified (as shown in the figure). This procedure is useful to avoid amplifying non-informative ligation products (e.g., self-ligation products and linear genome sequences that remained uncut by restriction enzymes). Note that the primer pair should be designed such that the PCR product contains the SNP site. The SNP site is denoted by a star sign. The resulting PCR product is gel-purified, and then subject to simultaneous fragmentation and adapter ligation via the Nextera DNA Sample Preparation Kit. The DNA library is sequenced on the MiSeq platform.

C) The generated reads are aligned to “modified genome” that is comprised of “artificial chromosome” and “masked reference genome”. The artificial chromosome is an exact sequence of the 3C PCR product in which the reverse complement of the fragment containing the promoter of a 9p21 gene and the fragment containing the SNP site are combined. The masked reference genome is hg19 in which the sequences corresponding to the restriction fragments are masked (i.e., replaced by Ns). The masked reference genome was used to avoid misalignments of non-specific sequences to the artificial chromosome. The reads aligned to the artificial chromosome were used for further analyses. The reads with low mapping quality (MAPQ<30) and the reads derived from PCR duplicates were filtered out. The presence of chromatin interactions is verified by interrogating the sequence reads that overlapped with the ligation junctions and the paired-end tag sequences that are aligned to both of the fragments containing the SNP site and the promoter. The number of paired-end read tag counts supporting reference and alternative alleles (i.e., allele-specific paired-end read tag counts) was measured by using The allele-specific paired-end tag counts were measured by using only high confidence base calls (base quality > 20) at the SNP site. The allele specific chromatin interaction is evaluated as the difference in contact frequencies between the two alleles in the mapped sequence reads at the SNP site by means of binomial test or likelihood ratio test as shown in Experimental Procedures.
