# Supplementary material for: Allelic Imbalance in Regulation of ANRIL through Chromatin Interaction at 9p21 Endometriosis Risk Locus
Source: PLoS Genet. 2016 Apr 7;12(4):e1005893. doi: 10.1371/journal.pgen.1005893 (PMC4824487; doi:10.1371/journal.pgen.1005893)
Supplement: S11 Fig — (PDF) [file pgen.1005893.s011.pdf]

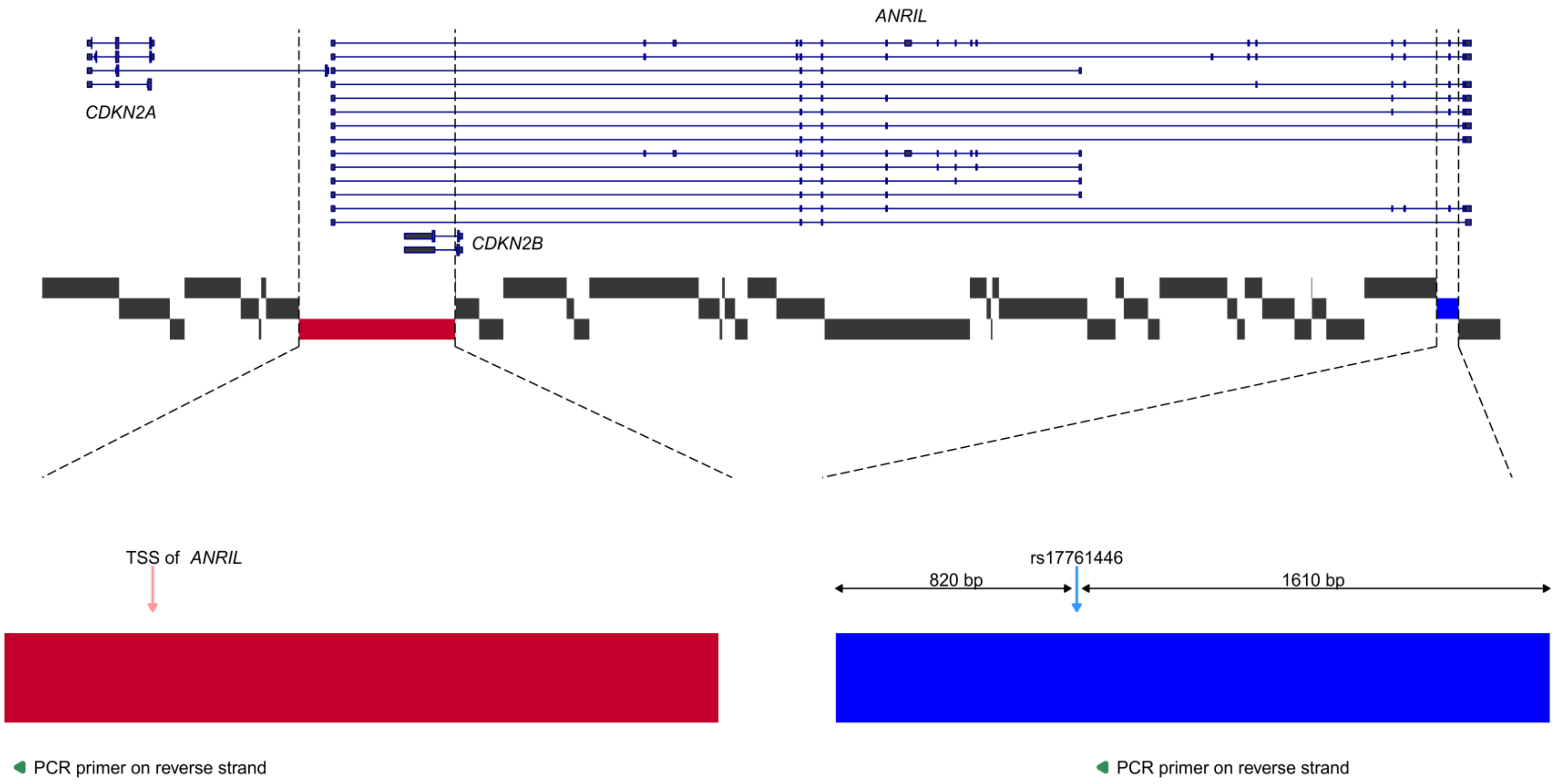

S11 Fig. Unidirectional primer design for the detection of chromatin interaction between fragment containing SNP rs17761446 and fragment containing the transcription start site (TSS) of *ANRIL*.
Gene structures of three 9p21 genes (top). Consecutive fragments around the three genes (middle). The fragments containing the SNP and TSS of *ANRIL* are color-coded by blue and red, respectively. The primer pair was set to be directed on the reverse strand (bottom).
