# Supplementary material for: Allelic Imbalance in Regulation of ANRIL through Chromatin Interaction at 9p21 Endometriosis Risk Locus
Source: PLoS Genet. 2016 Apr 7;12(4):e1005893. doi: 10.1371/journal.pgen.1005893 (PMC4824487; doi:10.1371/journal.pgen.1005893)
Supplement: S13 Fig — (PDF) [file pgen.1005893.s013.pdf]

TF	Sequence logo	Matched sequence	Score	
			Ref	Alt
CEBPE	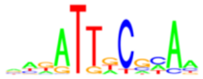	GATGTTTC[T/G]AAC	0.902	0.937
FOXP2	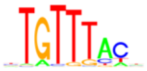	ATGTTTC[T/G]A	0.902	<0.85
HOXC8	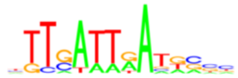	ATGTTTC[T/G]AACAAA	0.910	<0.85
LEF1	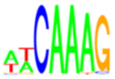	AACAAAG	0.997	0.997
PRGR	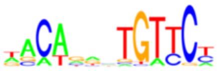	GGCAGATGTTTC[T/G]	0.904	<0.85
SOX4	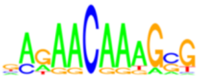	TC[T/G]AACAAAGGA	0.920	0.937
SOX5	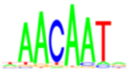	[T/G]AACAAAG	0.939	0.919
SOX9	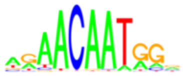	C[T/G]AACAAAGGA	0.901	0.931
SOX13	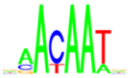	[T/G]AACAAAG	0.961	0.976
SOX15	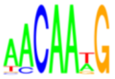	AACAAAG	0.993	0.993
SRY	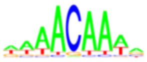	C[T/G]AACAAAG	0.900	0.876
TBX2	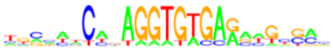	TTC[T/G]AACAAAGGAGAGAGTTGCCCA	0.905	0.904
TCF7	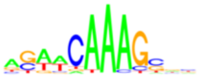	C[T/G]AACAAAGGAG	0.929	0.953
TCF7L2	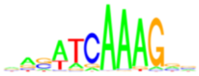	TC[T/G]AACAAAGGA	0.873	0.911
TEAD3	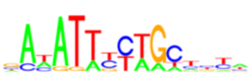	GATGTTTC[T/G]AACAAA	0.905	<0.85
UBIP1	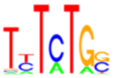	TTTC[T/G]AA	0.914	<0.85

S13 Fig. Transcription factor binding motif search around rs17761446.
Sequence logo was generated based on the position weight matrix from Hocomoco database. Scores for sequence with reference or alternative allele at the SNP site was calculated by MotifLocator. Transcription factors with score > 0.9 are shown.
