# Supplementary material for: Allelic Imbalance in Regulation of ANRIL through Chromatin Interaction at 9p21 Endometriosis Risk Locus
Source: PLoS Genet. 2016 Apr 7;12(4):e1005893. doi: 10.1371/journal.pgen.1005893 (PMC4824487; doi:10.1371/journal.pgen.1005893)
Supplement: S14 Fig — (PDF) [file pgen.1005893.s014.pdf]

TF	Sequence logo	Matched sequence	Score	
			Ref	Alt
KLF3	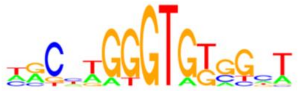	AGGATGGGTAG T/C GCT	0.944	0.874
NKX2-1	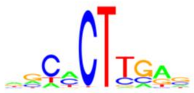	AG T/C GCTTGGA	0.902	0.870
YBX1	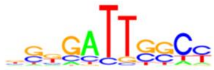	AG T/C GCTTGGAT	0.879	0.902
ZEP2	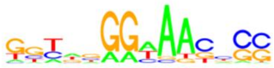	GGATGGGTAG T/C GCT	0.909	0.897
ZIC1	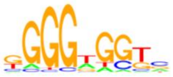	TGGGTAG T/C G	0.912	0.948
ZIC3	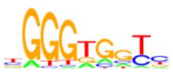	TGGGTAG T/C G	0.932	0.946

S14 Fig. Transcription factor binding motif search around rs17834457.
Sequence logo was generated based on the position weight matrix from Hocomoco database. Scores for sequence with reference or alternative allele at the SNP site was calculated by MotifLocator. Transcription factors with score > 0.9 are shown.
