# Supplementary material for: Allelic Imbalance in Regulation of ANRIL through Chromatin Interaction at 9p21 Endometriosis Risk Locus
Source: PLoS Genet. 2016 Apr 7;12(4):e1005893. doi: 10.1371/journal.pgen.1005893 (PMC4824487; doi:10.1371/journal.pgen.1005893)
Supplement: S15 Fig — (PDF) [file pgen.1005893.s015.pdf]

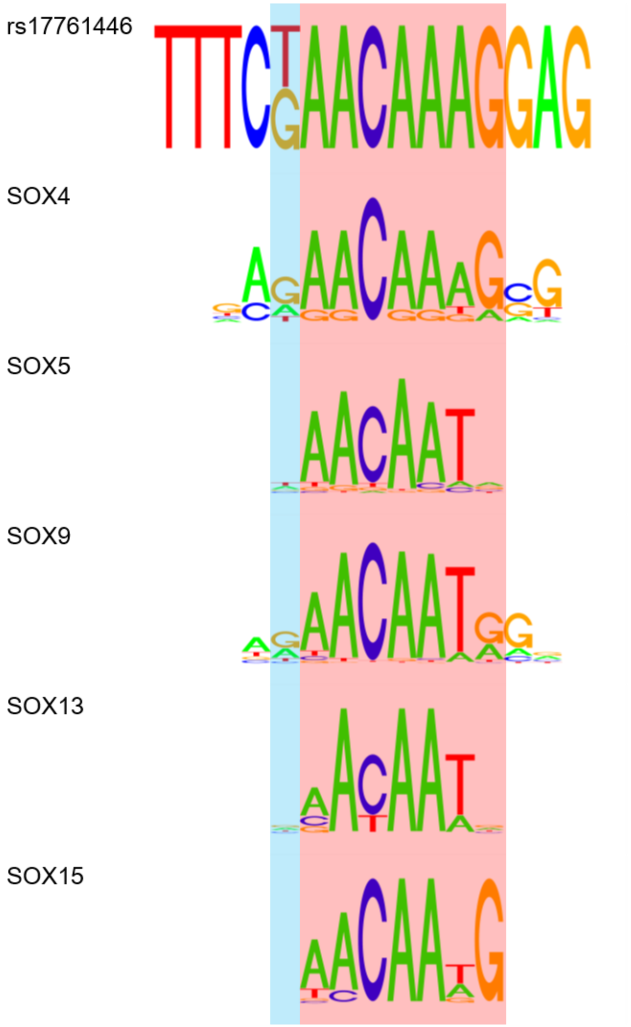

S15 Fig. Alignments of consensus motifs of SOX family to sequence surrounding rs17761446.

Core motif of HMG class of TFs are highlighted by red. The position of rs17761446 is highlighted by blue.
