# Supplementary material for: Allelic Imbalance in Regulation of ANRIL through Chromatin Interaction at 9p21 Endometriosis Risk Locus
Source: PLoS Genet. 2016 Apr 7;12(4):e1005893. doi: 10.1371/journal.pgen.1005893 (PMC4824487; doi:10.1371/journal.pgen.1005893)
Supplement: S16 Fig — (PDF) [file pgen.1005893.s016.pdf]

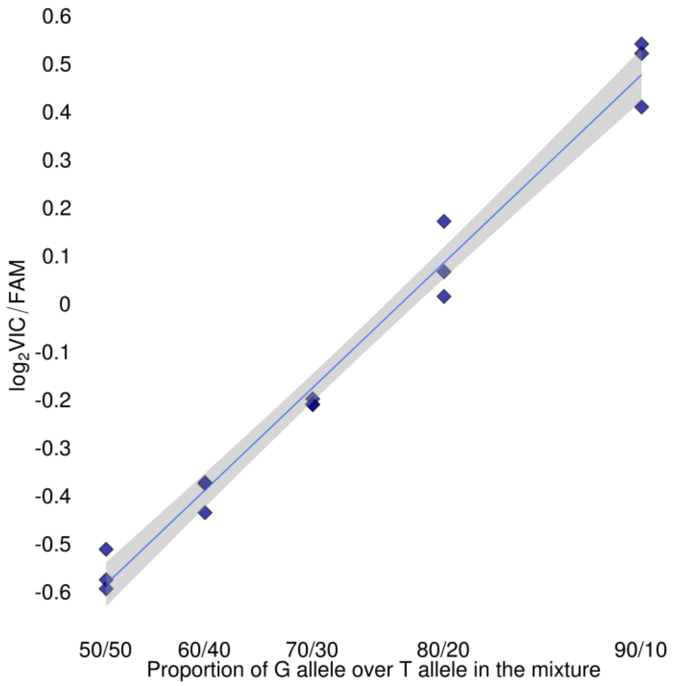

S16 Fig. Standard curve of the VIC/FAM ratios by TaqMan-based allelic discrimination assay.

Three pairs of DNA samples with GG and TT homozygous genotypes were mixed at the following proportions: 50:50, 60:40, 70:30, 80:20, and 90:10. The log2 transformed VIC/FAM ratios were regressed on the log2 transformed ratios of the two alleles. The curve showed a high level of linearity (Pearson's correlation coefficient: $r = 0.992$; $P = 5.3\times10^{-13}$).
