# Supplementary material for: Allelic Imbalance in Regulation of ANRIL through Chromatin Interaction at 9p21 Endometriosis Risk Locus
Source: PLoS Genet. 2016 Apr 7;12(4):e1005893. doi: 10.1371/journal.pgen.1005893 (PMC4824487; doi:10.1371/journal.pgen.1005893)
Supplement: S17 Fig — A) VIC/FAM ratios for immunoprecipitated and input chromatins by TaqMan allelic discrimination assay. B) Allelic ratios for ChIP libraries determined based on the standard curve. (PDF) [file pgen.1005893.s017.pdf]

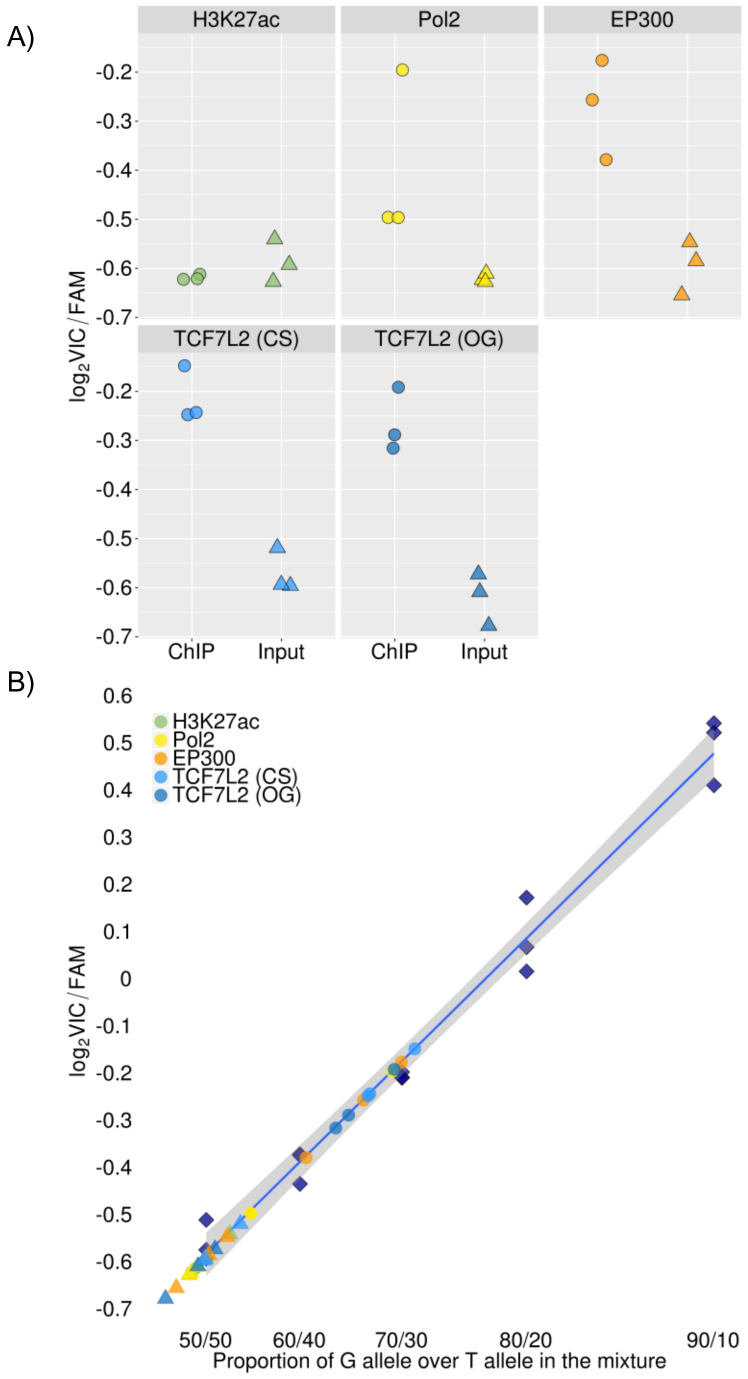

S17 Fig. Quantification of allele-specific factor bindings at rs17761446 based on the standard curve.
A) VIC/FAM ratios for immunoprecipitated and input chromatins by TaqMan allelic discrimination assay. ChIP assays in HEC251 were triplicated for each factor.
B) Allelic ratios for ChIP libraries were determined based on the standard curve created by using the mixture of DNA samples with known allelic ratios.
Pol2, RNA polymerase II; TCF7L2 (CS) and TCF7L2 (OG), TCF7L2 antibodies by Cell Signaling Technology and OriGene Technologies, respectively.
