# Supplementary material for: Allelic Imbalance in Regulation of ANRIL through Chromatin Interaction at 9p21 Endometriosis Risk Locus
Source: PLoS Genet. 2016 Apr 7;12(4):e1005893. doi: 10.1371/journal.pgen.1005893 (PMC4824487; doi:10.1371/journal.pgen.1005893)
Supplement: S18 Fig — (PDF) [file pgen.1005893.s018.pdf]

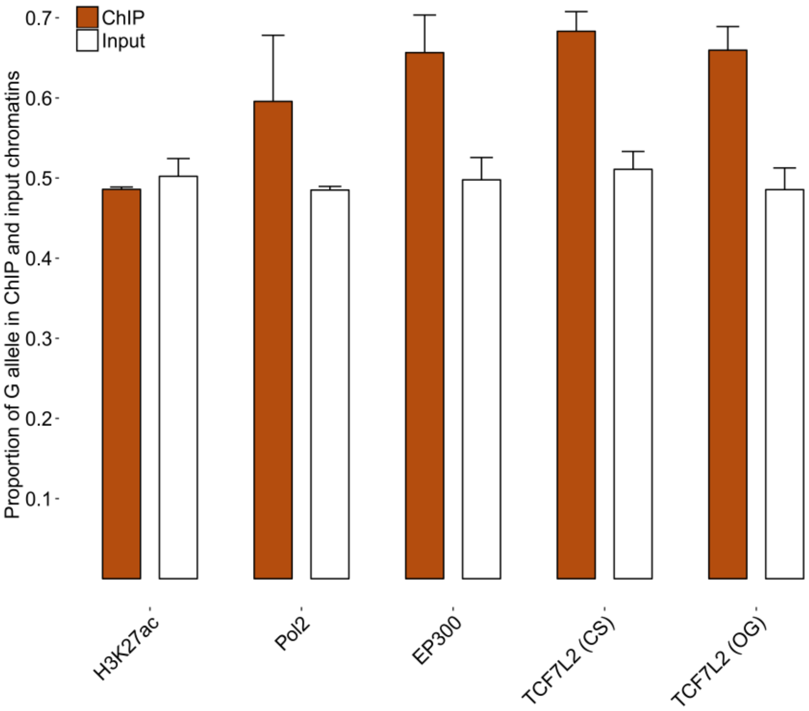

S18 Fig. Allele-specific factor bindings in HEC251 cells.
The proportions of G allele within immunoprecipitated (ChIP) and input chromatins. Data represents the mean and standard deviation of triplicated ChIP assays. Pol2, RNA polymerase II; TCF7L2 (CS) and TCF7L2 (OG), TCF7L2 antibodies by Cell Signaling Technology and OriGene Technologies, respectively..
