# Supplementary figures and images for: Allelic Imbalance in Regulation of ANRIL through Chromatin Interaction at 9p21 Endometriosis Risk Locus
Source: PLoS Genet. 2016 Apr 7;12(4):e1005893. doi: 10.1371/journal.pgen.1005893 (PMC4824487; doi:10.1371/journal.pgen.1005893)

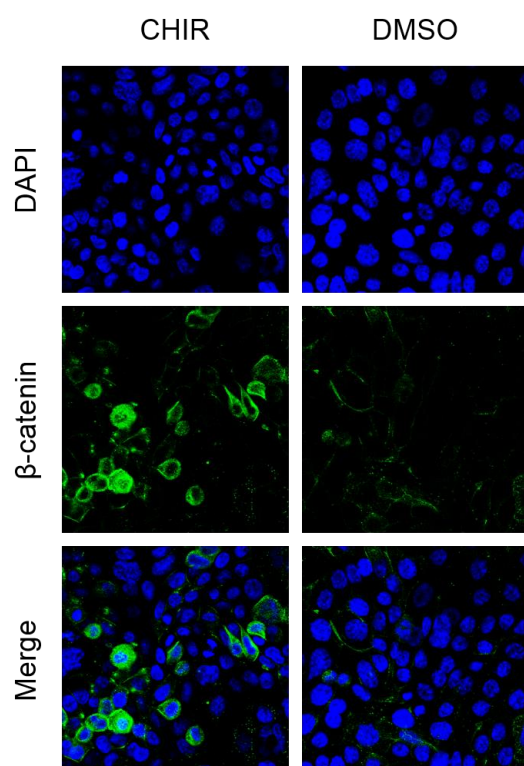

**S19 Fig. Immunofluorescence analysis for CHIR- and DMSO-treated HEC251 cells.**

Supplement: S19 Fig — (PDF) [file pgen.1005893.s019.pdf]
