# Supplementary material for: Allelic Imbalance in Regulation of ANRIL through Chromatin Interaction at 9p21 Endometriosis Risk Locus
Source: PLoS Genet. 2016 Apr 7;12(4):e1005893. doi: 10.1371/journal.pgen.1005893 (PMC4824487; doi:10.1371/journal.pgen.1005893)
Supplement: S20 Fig — A) Structures of ANRIL transcripts amplified by three primer pairs. B) Fold expressions of CHIR-treated cells for transcripts of ANRIL, CDKN2A and CDKN2B relative to cells treated with vehicle only (DMSO). (PDF) [file pgen.1005893.s020.pdf]

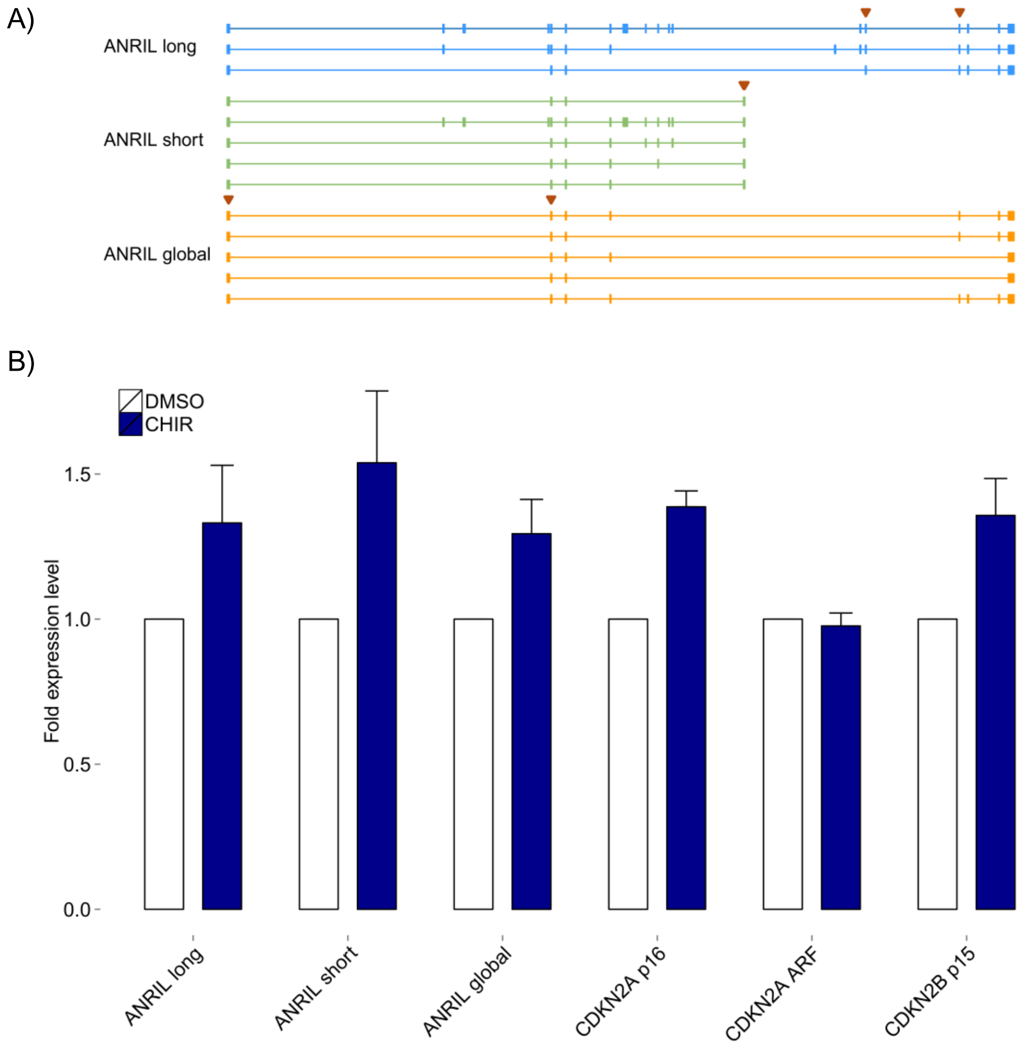

S20 Fig. Induction of Wnt signaling alters expression levels of 9p21 genes.
A) Structures of *ANRIL* transcripts amplified by three primer pairs. The positions of oligonucleotides primers are depicted by red arrow heads. The primer pair "*ANRIL* global" amplifies transcripts included in "*ANRIL* long" and "*ANRIL* short".
B) Fold expressions of CHIR-treated cells for transcripts of *ANRIL*, *CDKN2A* and *CDKN2B* relative to cells treated with vehicle only (DMSO). Data represent the mean and standard deviation of six experiments in HEC251 cells.
