# Supplementary material for: Allelic Imbalance in Regulation of ANRIL through Chromatin Interaction at 9p21 Endometriosis Risk Locus
Source: PLoS Genet. 2016 Apr 7;12(4):e1005893. doi: 10.1371/journal.pgen.1005893 (PMC4824487; doi:10.1371/journal.pgen.1005893)
Supplement: S21 Fig — (PDF) [file pgen.1005893.s021.pdf]

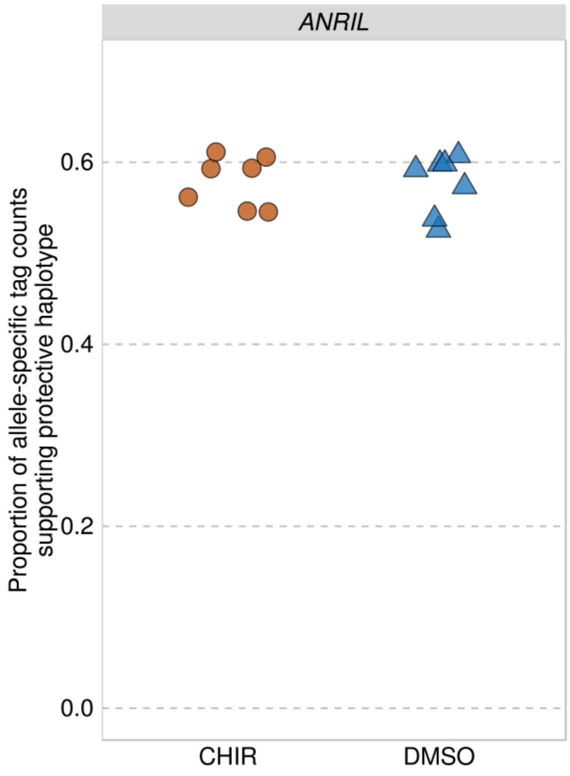

S21 Fig. ASE analysis of rs17761446 for *ANRIL* in CHIR- and DMSO-treated HEC251 cells.

A SNP in transcribed region in *ANRIL* (rs10965215) are examined. For each treatment, results of RNA-seq for seven experiments are plotted.
