# Supplementary material for: Allelic Imbalance in Regulation of ANRIL through Chromatin Interaction at 9p21 Endometriosis Risk Locus
Source: PLoS Genet. 2016 Apr 7;12(4):e1005893. doi: 10.1371/journal.pgen.1005893 (PMC4824487; doi:10.1371/journal.pgen.1005893)
Supplement: S22 Fig — (PDF) [file pgen.1005893.s022.pdf]

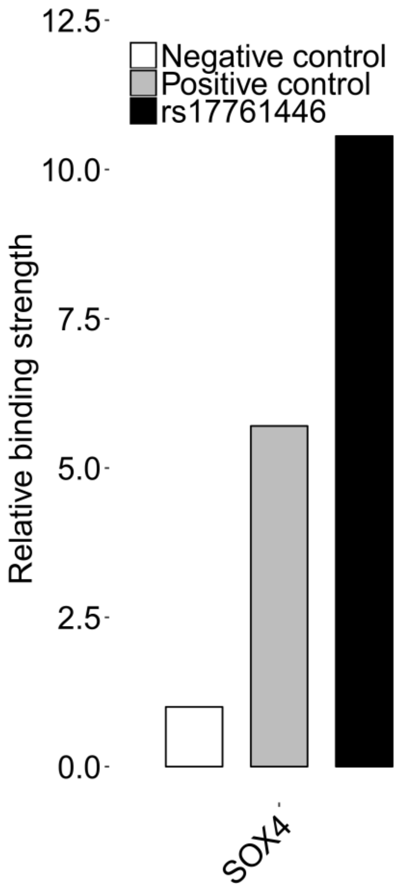

S22 Fig. ChIP assays for SOX4 at rs17761446 site in HEC251 cells.
MYC promoter and α satellite repeat element were used as positive and negative control regions, respectively. The enrichment of the normalized binding affinity for the candidate SNP site or positive control region was normalized by dividing by that for negative control region.
