# Supplementary material for: Allelic Imbalance in Regulation of ANRIL through Chromatin Interaction at 9p21 Endometriosis Risk Locus
Source: PLoS Genet. 2016 Apr 7;12(4):e1005893. doi: 10.1371/journal.pgen.1005893 (PMC4824487; doi:10.1371/journal.pgen.1005893)
Supplement: S23 Fig — (PDF) [file pgen.1005893.s023.pdf]

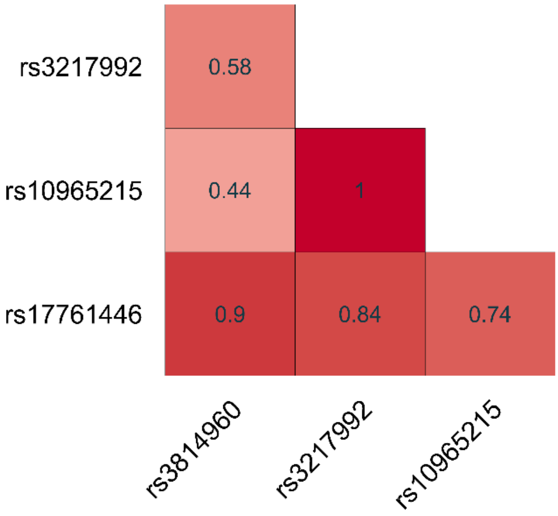

S23 Fig. LD structure for SNPs used in ASE analyses.
D’ values for each pair of SNP are shown. rs17761446 is a candidate causal SNP. rs3814960, rs3217992, and rs10965215 are transcribed SNPs within *CDKN2A*, *CDKN2B*, and *ANRIL*, respectively.
