# Supplementary material for: Allelic Imbalance in Regulation of ANRIL through Chromatin Interaction at 9p21 Endometriosis Risk Locus
Source: PLoS Genet. 2016 Apr 7;12(4):e1005893. doi: 10.1371/journal.pgen.1005893 (PMC4824487; doi:10.1371/journal.pgen.1005893)
Supplement: S1 Table — (PDF) [file pgen.1005893.s024.pdf]

S1 Table. Variants exhibiting strong LD with two SNPs identified by the original GWASs.

Variant	GWAS lead SNP	Position	Type	Novelty	Ref	Alt	r^2	D’
rs17761197	rs10965235	22072730	SNV	known	T	C	0.861	0.928
rs138681154	rs10965235	22074065	SNV	known	A	T	0.861	0.928
rs7855162	rs10965235	22074793	SNV	known	C	T	0.861	0.928
rs77568230	rs10965235	22075042	SNV	known	T	A	0.861	0.928
rs57076710	rs10965235	22075459	SNV	known	G	T	0.8	0.926
rs78789397	rs10965235	22082094	SNV	known	G	A	0.861	0.928
rs79896137	rs10965235	22083831	SNV	known	A	G	0.861	0.928
rs75221032	rs10965235	22086767	SNV	known	A	T	0.929	1
rs80076828	rs10965235	22087604	SNV	known	C	A	1	1
rs78651848	rs10965235	22087688	SNV	known	G	A	1	1
rs80307048	rs10965235	22089264	SNV	known	A	G	1	1
rs79331368	rs10965235	22099223	SNV	known	T	C	1	1
rs74772332	rs10965235	22102464	SNV	known	A	G	1	1
rs141403575	rs10965235	22105584	indel	known	-	AG	1	1
rs17761319	rs10965235	22110478	SNV	known	G	T	1	1
rs17834367	rs10965235	22112606	SNV	known	C	T	1	1
rs77563194	rs10965235	22114726	SNV	known	T	C	1	1
rs10965234	rs10965235	22115078	SNV	known	T	G	1	1
rs944796	rs10965235	22115285	SNV	known	G	C	1	1
rs59995170	rs10965235	22115949	SNV	known	A	G	1	1
rs78264155	rs10965235	22116247	SNV	known	T	C	1	1
rs17834457	rs10965235	22118026	SNV	known	T	C	1	1
rs17761446	rs10965235	22118102	SNV	known	G	T	1	1
rs78766516	rs10965235	22120371	SNV	known	A	G	1	1
rs34843542	rs10965235	22121378	indel	known	T	-	1	1
rs17761458	rs10965235	22124368	SNV	known	G	A	1	1
rs6475610	rs1537377	22141894	SNV	known	T	C	0.814	0.902
rs6475612	rs1537377	22152580	SNV	known	A	G	0.859	0.95
rs5896970	rs1537377	22170023	indel	known	-	C	0.908	1
rs828582	rs1537377	22172259	SNV	known	A	T	0.87	1
rs10811669	rs1537377	22172893	SNV	known	C	T	0.954	1
rs9987548	rs1537377	22173075	SNV	known	A	T	0.954	1

rs1981046	rs1537377	22173407	SNV	known	C	T	0.87	1
rs9987689	rs1537377	22173676	SNV	known	G	A	0.859	0.95
