# Supplementary material for: Allelic Imbalance in Regulation of ANRIL through Chromatin Interaction at 9p21 Endometriosis Risk Locus
Source: PLoS Genet. 2016 Apr 7;12(4):e1005893. doi: 10.1371/journal.pgen.1005893 (PMC4824487; doi:10.1371/journal.pgen.1005893)
Supplement: S2 Table — (PDF) [file pgen.1005893.s025.pdf]

S2 Table. Oligonucleotides used in 3C.

Type	Enzyme	Feature	Fragment	Size	Primer	Primer position	Strand	Distance*
**Initial screening step**								
Target	*Eco*RI		21967114 – 21971393	4280	CGGAAAGGAAGCTTGTGTAGAG	21971256 – 21971277	+	116
Target	*Eco*RI	TSS of *CDKN2A*	21971394 – 21975688	4295	CCGAGAATCGAAATCACCTGTA	21975426 – 21975447	+	241
Target	*Eco*RI		21975689 – 21977823	2135	CGGCCTACAGTGGTTCTTAAT	21977660 – 21977680	+	143
Target	*Eco*RI		21977824 – 21981382	3559	CTTGAGTGGAGCCTACAGTAATC	21981251 – 21981273	+	109
Target	*Eco*RI		21981383 – 21984218	2836	GCACGGTAGGCTCTCAATAAA	21984025 – 21984045	+	173
Target	*Eco*RI		21984219 – 21991018	6800	CTGAATGAAAGTGGATGGGTTC	21990887 – 21990908	+	110
Target	*Eco*RI	TSS of *ANRIL*	21991019 – 21998162	7144	TTTCTCTCCACAGGAACTAGAC	21997962 – 21997983	+	179
Target	*Eco*RI		21998163 – 22001072	2910	TCCTGACTCCTACTCTGTTATCC	22000917 – 22000939	+	133
Target	*Eco*RI		22001073 – 22007886	6814	TAGCTGCTGTTAGTATTTCAGA	22007741 – 22007762	+	124
Target	*Eco*RI		22007887 – 22008414	528	TGAGGAATCCCGTCTCATTCT	22008303 – 22008323	+	91
Target	*Eco*RI	TSS of *CDKN2B*	22008415 – 22009323	909	CCTGGATTGCTTCTGGGAAA	22009186 – 22009205	+	118
Target	*Eco*RI		22009324 – 22011162	1839	GCGAAGCAAGTTGACTGAATG	22011030 – 22011050	+	112
Target	*Eco*RI		22011163 – 22013536	2374	GTCTCTTGTATCTCTCTCTCTCTCT	22013427 – 22013451	+	85
Target	*Eco*RI		22013537 – 22017423	3887	AAGGAAGTCTGCCTATATGGGTT	22017350 – 22017372	+	51
Target	*Eco*RI		22017424 – 22017712	289	CTTGTGTACCTTGTCACATCAGTAA	22017625 – 22017649	+	63
Anchor	*Eco*RI	rs17761446	22114805 – 22122022	7218	TTGGATGCCTGGATTGATTCTA	22121798 – 22121819	+	203
**AS3C-seq**								
Target	*Hin*dIII	TSS of *ANRIL*	21991197 – 22008507	17311	AGTCCTTGGAGAAACAGAGAAA	21991488 – 21991509	-	291
Anchor	*Hin*dIII	rs17761446	22117283 – 22119713	2431	GGTGACTATGTGGTGCAACT	22118179 – 22118198	-	896

* Distance between primer and closer restriction enzyme cleaved site.
