# Supplementary material for: Allelic Imbalance in Regulation of ANRIL through Chromatin Interaction at 9p21 Endometriosis Risk Locus
Source: PLoS Genet. 2016 Apr 7;12(4):e1005893. doi: 10.1371/journal.pgen.1005893 (PMC4824487; doi:10.1371/journal.pgen.1005893)
Supplement: S3 Table — (PDF) [file pgen.1005893.s026.pdf]

S3 Table. PCR primers for SNP genotyping, ChIP assay and gene expression analysis.

Target	Forward	Reverse	Reference
**SNP genotyping**			
rs10965235	CCTGCTTTCAAGAGCCATGT	TAAGCCACCAAGGAAGAGGA	Primer3Plus[1]
rs17761446_geno	CAAGCTCCCGGTTAGAGTGA	ATGGCTCATGACAAGGGAGC	Primer3Plus[1]
**ChIP assay**			
rs17761446_chip	GCCTTCTTGGCAGATGTTTC	GTGAGCCTATTCGCACAGCT	Primer3Plus[1]
c-myc promoter	CCCAAAAAAAGGCACGGAA	TATTGGAAATGCGGTCATGC	Millipore Cat.# CS200601
α satellite repeat	Not available	Not available	Cell Signaling Cat. # 4486
**Gene expression**			
ACTB	GTACCACTGGCATCGTGATGGACT	CCGCTCATTGCCAATGGTGAT	
ANRIL (long)[*]	GGAATGAGGAGCACAGTGAT	CCAGTGGTGGATGTTGTAGA	Jarinova et al.[2]
ANRIL (short)[†]	GGAGCTAGGAATTTCCTACG	CAAGATAGAGAAGCAGGTATC	Jarinova et al. [2]
ANRIL (global)[‡]	CAATCAGGAGGCTGAATGTC	TGCTGTTGAATCAGAATGAGG	Jarinova et al. [2]
CDKN2A (*p16*)	CACCGAATAGTTACGGTCGG	GCACGGGTCGGGTGAGAGTG	Kotake et al.[3]
CDKN2A (*ARF*)	GGCCCTCGTGCTGATGCTAC	TGGAGCAGCAGCAGCTCCGC	Kotake et al.[3]
CDKN2B (*p15*)	GGTGGCTACGAATCTTCCG	CCTAAGTTGTGGGTTCACCA	Jarinova et al. [2]
**Allele-specific expression**			
ANRIL (rs10965215, RNA)	TGCTCTATCCGCCAATCAGG	AATAGGTGTGGGCCTCAGTG	Primer3Plus[1]
ANRIL (rs10965215, DNA)	AGCCTGATCATGTGTGGTCTG	TGATGGTTTCCCAAACAGCAC	Primer3Plus[1]
CDKN2A (rs3814960, RNA)	ACTTCAGGGGTGCCACATTC	GGCCTCCGACCGTAACTATT	Primer3Plus[1]
CDKN2A (rs3814960, DNA)	ACTTCAGGGGTGCCACATTC	GGCCTCCGACCGTAACTATT	Primer3Plus[1]
CDKN2B (rs3217992, RNA)	TGTAAGCTCCTAAGGTGGCT	CCAGGTGGCTTCGAAAATGG	Primer3Plus[1]
CDKN2B (rs3217992, DNA)	TGTAAGCTCCTAAGGTGGCT	CCAGGTGGCTTCGAAAATGG	Primer3Plus[1]

[*] The primer pair target for long transcript of *ANRIL* (RefSeq Accession: NR_003529, NR_047532, NR_047534).
[†] The primer pair target for short transcripts of *ANRIL* (NR_047533, NR_047539, NR_047540, NR_047541, and NR_047542).
[‡] The primer pair target for both long (NR_003529, NR_047532, NR_047534, NR_047535, NR_047536, NR_047537, NR_047538, and

NR_047543) and short (NR_047533, NR_047539, NR_047540, NR_047541, and NR_047542) transcripts of *ANRIL*.

1. Untergasser A, Nijveen H, Rao X, Bisseling T, Geurts R, Leunissen JA. Primer3Plus, an enhanced web interface to Primer3.Nucleic Acids Res. 2007. W71-4.
2. Jarinova O, Stewart AF, Roberts R, Wells G, Lau P, Naing T, et al. Functional analysis of the chromosome 9p21.3 coronary artery disease risk locus. Arterioscler Thromb Vasc Biol. 2009. 1671-7.
3. Kotake Y, Cao R, Viatour P, Sage J, Zhang Y, Xiong Y. pRB family proteins are required for H3K27 trimethylation and Polycomb repression complexes binding to and silencing p16INK4alpha tumor suppressor gene. Genes Dev. 2007. 49-54.
