# Supplementary material for: Allelic Imbalance in Regulation of ANRIL through Chromatin Interaction at 9p21 Endometriosis Risk Locus
Source: PLoS Genet. 2016 Apr 7;12(4):e1005893. doi: 10.1371/journal.pgen.1005893 (PMC4824487; doi:10.1371/journal.pgen.1005893)
Supplement: S4 Table — (PDF) [file pgen.1005893.s027.pdf]

S4 Table. Antibodies used in ChIP and immunofluorescence analyses.

Factor	Host	Antibody type	Provider	Cat. No.
TCF7L2	Rabbit	Monoclonal	Cell Signaling	2565
TCF7L2	Rabbit	Polyclonal	OriGene	TA590280
EP300	Rabbit	Polyclonal	Santa Cruz	Sc-584
RNA polymerase II	Rabbit	Polyclonal	Abcam	ab5131
H3K27ac	Rabbit	Polyclonal	Abcam	ab4729
SOX4	Goat	Polyclonal	Santa Cruz	Sc-17326
β catenin	Rabbit	Polyclonal	Abcam	Ab8480

Normal rabbit IgG (Cell Signaling, 2729) was used as negative control in ChIP assay.
